# Arachidonic acid impairs natural killer cell functions by disrupting signaling pathways driven by activating receptors and reactive oxygen species

**DOI:** 10.1186/s12964-024-01940-z

**Published:** 2024-11-19

**Authors:** Mohamad K. Hammoud, Celina Meena, Raimund Dietze, Nathalie Hoffmann, Witold Szymanski, Florian Finkernagel, Andrea Nist, Thorsten Stiewe, Johannes Graumann, Elke Pogge von Strandmann, Rolf Müller

**Affiliations:** 1 Department of Translational Oncology, Center for Tumor Biology and Immunology, Philipps University, Marburg, Germany; 2 Institute of Physiological Chemistry, Philipps University, Marburg, Germany; 3 Institute of Tumor Immunology, Center for Tumor Biology and Immunology, Philipps University, Marburg, Germany; 4 Institute of Translational Proteomics, Biochemical/Pharmacological Centre, Philipps University, Marburg, Germany; 5 Core Facility Translational Proteomics, Philipps University, Marburg, Germany; 6 Genomics Core Facility, Philipps University, Marburg, Germany; 7 Center for Tumor Biology and Immunology (ZTI), Philipps University, Hans-Meerwein-Strasse 3, 35043 Marburg, Germany

**Keywords:** Arachidonic acid, Cytotoxicity receptors, Interleukin 2, Natural killer (NK) cells, NKG2D, Ovarian carcinoma, Phosphoproteomics, Reactive oxygen species, STAT1

## Abstract

**Background:**

High levels of the polyunsaturated fatty acid arachidonic acid (AA) within the ovarian carcinoma (OC) microenvironment correlate with reduced relapse-free survival. Furthermore, OC progression is tied to compromised immunosurveillance, partially attributed to the impairment of natural killer (NK) cells. However, potential connections between AA and NK cell dysfunction in OC have not been studied.

**Methods:**

We employed a combination of phosphoproteomics, transcriptional profiling and biological assays to investigate AA’s impact on NK cell functions.

**Results:**

AA (i) disrupts interleukin-2/15-mediated expression of pro-inflammatory genes by inhibiting STAT1-dependent signaling, (ii) hampers signaling by cytotoxicity receptors through disruption of their surface expression, (iii) diminishes phosphorylation of NKG2D-induced protein kinases, including ERK1/2, LYN, MSK1/2 and STAT1, and (iv) alters reactive oxygen species production by transcriptionally upregulating detoxification. These modifications lead to a cessation of NK cell proliferation and a reduction in cytotoxicity.

**Conclusion:**

Our findings highlight significant AA-induced alterations in the signaling network that regulates NK cell activity. As low expression of several NK cell receptors correlates with shorter OC patient survival, these findings suggest a functional linkage between AA, NK cell dysfunction and OC progression.

**Supplementary Information:**

The online version contains supplementary material available at 10.1186/s12964-024-01940-z.

## Abstract (plain language summary)

High levels of the fatty acid arachidonic acid (AA) in the ovarian cancer environment are associated with shorter survival. Ovarian cancer progression is linked to weakened immune defenses, partly due to malfunctioning natural killer (NK) cells. We used various scientific methods to study how AA affects NK cell function. Our results show that AA significantly changes the signaling processes that control NK cell activity. Specifically, AA (i) disrupts the expression of pro-inflammatory genes, (ii) impairs the function of receptors that activate killing mechanisms by preventing them from appearing on the cell surface, (iii) reduces the activity of intracellular signaling proteins that are activated by these receptors and (iv) changes the production of reactive oxygen species. These changes stop NK cells from multiplying and reduce their ability to kill cancer cells, suggesting that AA contributes to NK cell dysfunction and cancer progression.

**Supplementary Information:**

The online version contains supplementary material available at 10.1186/s12964-024-01940-z.

## Introduction

NK and innate lymphocyte 1 cells [1] represent critical components of the innate immune system and play an important role in the elimination of cancer and infected cells through a range of mechanisms [2–4]. Using activating and inhibitory receptors, they distinguish healthy from tumor cells [5]. Activating NK cell receptors, for instance, bind stress-induced ligands selectively produced by cancer cells. They include (i) Natural Killer Group 2, Member D (NKG2D), which recognizes stress-induced ligands (MICA/B, ULBPs) on tumor cells; (ii) Natural Cytotoxicity Receptors (NCRs), including NKp30, NKp44, and NKp46, which also recognize diverse ligands on tumor cells and (iii) CD16 (FcγRIII), which triggers antibody-dependent cellular cytotoxicity (ADCC) by binding to the Fc domain of opsonizing antibodies bound to tumor cells [5]. When activated, these receptors trigger the release of cytotoxic granules (including perforin and granzymes) and express death ligands such as tumor necrosis factor (TNF), TNFSF10 (TRAIL) and FASLG (FAS ligand; CD95L) to induce apoptosis [6]. A second relevant mechanism of the NK-cell-mediated anti-tumor response is the secretion of cytokines and chemokines [7, 8], in particular interferon-gamma (IFNγ), which can increase antigen presentation and activate other immune cells. NK cells also recruit other immune cells to the tumor site by secreting chemokines, such as MIP-1α, MIP1-β and RANTES, thereby potentially amplifying the anti-tumor immune response [9].

The tumor microenvironment (TME), however, suppresses NK cell activity through various mechanisms, especially at later stages of tumor progression. These suppressive mechanisms include inhibitory cytokines [10] as well as the shedding of NK cell activating ligands from the tumor cell surface, which blocks activating NK cell receptors, such as NKG2D [11–13]. Consistent with this inhibitory function, soluble NKG2D ligands in the TME are associated with a poor clinical outcome [14, 15]. Moreover, mediators of the TME, such as TGFβ and macrophage migration-inhibitory factor, inhibit NKG2D expression, thereby further suppressing NK cell activation [16–18]. In addition, extracellular vesicles released by OC cells with high surface expression of NKG2D ligands downregulate the expression of NKG2D receptors and their cytotoxicity [19].

Other phenotypic alterations are associated with the downregulation of cytotoxic receptors including CD16 on tumor-associated NK cells (TANK) from OC ascites, resulting in diminished CD16-dependent tumor cell killing (Vyas et al., 2017). This phenotype was also observed with tumor infiltrating NK cells in the OC TME, which additionally exhibited an exhaustion phenotype characterized by increased PD-1 expression [20].

OC cells release TGF-β, IL-10 as well as other immunosuppressive cytokines and express immune checkpoint molecules (e.g., PD-L1). These molecules directly inhibit NK cell function, but may also affect cellular interactions mandatory for the development of an antitumor immune response. One example is the cross-talk between activated NK cells and dendritic cells (DCs), which results in the recruitment of CD8^+^ effector T cells to the TME through upregulation of the CXCR3 and CCR5 ligands CXCL9, CXCL10 and CCL5 on DCs [21].

Healthy cells are protected from NK cell attack by a set of inhibitory receptors that recognize self-defining major histocompatibility complex (MHC) class I molecules [22]. These inhibitory receptors include (i) Killer Cell Immunoglobulin-like Receptors (KIRs), (ii) the CD94/NKG2A Complex and (iii) Leukocyte Immunoglobulin-like Receptors (LIRs). In order to evade T cells, transformed cells may not express these MHC molecules, which renders them susceptible to killing by NK cells. However, this “missing-self” mechanism frequently fails for cancer cells due to maintenance of MHC class I molecules, expression of non-classical MHC molecules that can engage inhibitory receptor or the suppression of activation signals, by for example, soluble NKG2D ligands or immunosuppressive cytokines [23–25].

The balance between signals from activating and inhibitory receptors determines whether the cytotoxic properties of NK cells are activated [26]. In the encounter of cancer cells, this balance is frequently skewed towards inhibitory mechanism, which represents a major determinant of inactivating the tumor-suppressive function of NK cells. Finally, NK cells express receptors for various cytokines produced by macrophages and other immune cell types that regulate their proliferation, survival and activation [7, 27]. Functionally crucial cytokines in this context are IL-2, IL-12, IL-15, IL-21 and IFNγ, whose synthesis is frequently suppressed in the TME, for example in ovarian carcinoma (OC) [28].

OC, and in particular its most frequent and aggressive subtype high-grade serous carcinoma, is the most lethal of all gynecological cancers with unmet therapeutic needs [29]. A hallmark of OC is its TME, which is composed of anatomically and functionally different compartments: solid tumor masses and the peritoneal fluid, which frequently occurs as ascites at advanced stages [30, 31] and mediates the metastatic spread of OC in the peritoneal cavity [28, 31–34]. Besides heterogeneous populations of cancer cells, immune cells represent key components of OC ascites, with T cells, NK cells and macrophages as dominant cell types. The interaction between these immune cells and cancer cells plays a critical role in immune suppression, tumor growth and therapy resistance [28, 31–34]. Non-cellular components playing a pivotal role in this context include a remodeled extracellular matrix, soluble factors, including cytokines, chemokines and growth factors, as well as extracellular vesicles carrying proteins, lipids and nucleic acids [19, 35]. These mediators produced by, and acting on, tumor cells, immune cells and other host cells establish an intricate network of reciprocal interactions that promote immune suppression and tumor progression. Notably, the signaling network of tumor and immune cells from ascites closely resembles that of metastatic lesions in the omentum [35].

One of the mediators dysregulated in the TME and linked to poor survival of OC is the polyunsaturated fatty acid (PUFA) arachidonic acid (AA). AA exerts its effects either indirectly after cyclooxygenase-catalyzed conversion to prostanoids, in particular prostaglandin E_2_ [36, 37], or by direct mechanisms, which remain only partially understood. We have previously reported that AA interferes with the function of macrophages in the TME by two fundamentally different mechanisms: (i) induction of Ca^2+^-dependent p38 pathway linked to deregulation of RHO-GTPases, impaired actin filament organization and augmented release of exosomes [38], and (ii) impairment of pro-inflammatory signal transduction due to impaired JAK/STAT signaling caused by AA-mediated perturbation of lipid rafts, which are crucial for the spatial organization of cytokine receptors and associated signaling proteins in the plasma membrane [39]. Notably, the first mechanism is triggered by AA at concentrations as low as 5 µM [38], whereas the disruption of lipid rafts occurs at significantly higher AA concentrations, around 50 µM [39]. Importantly, AA concentrations exceeding 50 µM are observed in a subset of OC patients and correlate with poorer clinical outcomes [40].

AA levels in the TME [40] as well the impairment of NK cell functions [14, 41, 42] are associated with an adverse clinical outcome of OC. Given those observations and the pivotal role of NK cells discussed above, we set out to investigate the impact of AA on NK cell function.

## Materials and methods

### Patient samples

Ascites was collected from ovarian high-grade serous carcinoma patients during first-line surgery at the University Hospital in Marburg. The acquisition and analysis of ascites and ascites-associated NK cells were approved by the local ethics committee (reference number 205/10). Donors provided their written consent in accordance with the Declaration of Helsinki.

### Cell lines

NK92, NKL and K562 cell lines were obtained from the American Type Culture Collection (Manassas, VA, USA) and OVCAR-8 cells from the NIGMS Human Genetic Cell Repository of the NIH (Bethesda, MD, USA). NK92 cell cultures were maintained in RPMI medium (#61870044, Gibco™, Thermo Fisher Scientific, Darmstadt, Germany) supplemented with 10% horse serum (#16050122; Gibco™), 10% fetal bovine serum (FBS; #10270106; Gibco™), 2 mM glutamine (#35050061, Gibco™), 1 mM sodium pyruvate (11-360-070; Gibco™), 1× nonessential amino acids (#11140050, Gibco™), 1% Penicillin-Streptomycin (P0781, Sigma-Aldrich, Taufkirchen, Germany), and 200 IU/mL IL-2 (#11340025; ImmunoTools, Friesoythe, Germany). K562 cells were cultured in DMEM (#41966052, Gibco™) medium supplemented with 5% FBS and 1% Penicillin-Streptomycin. NK92 cells were kept in 2.5% FBS for 2 h prior to stimulation with cytokines. OVCAR-8 cells were cultured in RPMI 1640 (#61870044, Thermo Fisher Scientific) supplemented with 10% FBS.

Peripheral blood mononuclear cells (PBMCs) were enriched by Ficoll density gradient centrifugation from Leukoreduction System (LRS) chambers from healthy adult volunteers kindly provided by the Center for Transfusion Medicine and Hemotherapy at the University Hospital Gießen and Marburg, as approved by the local ethics committee (205/10). NK cells were then separated from leukocytes by negative selection (NK Cell Isolation Kit, #130-092-657; Miltenyi Biotec, Bergisch Gladbach, Germany) following the instruction of the manufacturer. Isolated NK cells were cultured in RPMI medium with 10% FBS and 10 U/mL IL-2, and kept at 37 °C overnight.

### Chemicals

AA was purchased from Cayman Chemicals (Hamburg, Germany), dissolved at a concentration of 50 mM in ethanol (stock solution) and stored at -20 °C. A corresponding volume of ethanol was added to cell cultures as solvent control.

### Sample preparation for phosphoproteomics

NK92 and NKL cells (5 × 10^8^) were serum-starved for 24 h prior to treatment with 50 µM AA or solvent for 15 min. Cells were harvested by centrifugation at 300 x g for 5 min and lysed in 4% SDS, 100 mM Tris, pH 7.6 supplemented with Roche PhosSTOP (#4906845001; Sigma-Aldrich) and complete protease inhibitor cocktail (P8340, Sigma Aldrich). NK92 and NKL control and arachidonic acid-induced cells were subjected to proteomic profiling in quadruplicates at the Core Facility Translational Proteomics of the Department of Medicine at Philipps University Marburg. In brief, Na-Deoxycholate (Carl Roth, Karlsruhe, Germany) was added to lysates to a final concentration of 0.02%, followed by incubation at RT for 15 min. TCA was then added to 10% final concentration, followed by a further 1 h incubation at RT. Subsequently, samples were centrifuged at 4 °C for 10 min at 21,000 g, and the pellet retained. The pellet was washed with 1 mL of ice-cold acetone, followed by a further centrifugation at 4 °C for 10 min at 21,000 g, and pellet retention. Next, the pellet was resuspended in 250 ul of 4% N-lauroylsarcosine sodium salt (Sigma-Aldrich; 2% final concentration) in 50 mM TEAB buffer (Sigma-Aldrich). Following protein content estimation using BCA (Thermo Fisher Scientific), approximately 200 µg of protein were reduced and alkylated by addition of DTT to a final concentration of 10 mM and incubation at 95 °C for 10 min plus iodoacetamide to a final concentration of 13 mM and incubation for 30 min at RT in the dark. A modified version of the SP3 method [43] was used for further processing on an in-house-made magnetic rack. Protein binding was performed in a final concentration of 70% anhydrous acetonitrile (ACN) at neutral pH, followed by washes with 70% ethanol and 100% anhydrous acetonitrile. After acetonitrile removal, beads were resuspended in 200 µl 50 mM TEAB buffer, and 4 ug of trypsin (Promega, Madison, Wisconsin, USA) was added. Protein digestion was performed overnight, at 37 °C with shaking. Peptide concentration was estimated using the fluorimetric Pierce Quantitative Peptide Assays. From each sample, 100 µg peptides were used for TMT labeling, and volumes adjusted to 100 µl. Labeling efficiency of individual channels was mass spectrometrically checked to exceed 98%. As amine-directed TMT labeling interferes with precise peptide quantitation using the fluorimetic peptide assay, downstream sample amounts are expressed in relative sample volume of the respective preparatory step. A reference channel sample was created by mixing six randomly chosen samples from the set to a final peptide amount of 200 µg and split into two aliquots.

Two separate TMT mixes were designed according to the following scheme: TMT-sample_1 (TMTpro-127 C - NK92_Control rep1, TMTpro-128 C - NK92 Control rep2, TMTpro-129 C - NK92 Arachidonic acid rep1, TMTpro-130 C - NK92 Arachidonic acid rep2, TMTpro-131 C - NKL Control rep1, TMTpro-132 C - NKL Control rep2, TMTpro-133 C - NKL Arachidonic acid rep1, TMTpro-134 C - NKL Arachidonic acid rep2, TMTpro-135 N - reference), TMT-sample_2 (TMTpro-127 C - NK92_Control rep3, TMTpro-128 C - NK92 Control rep4, TMTpro-129 C - NK92 Arachidonic acid rep3, TMTpro-130 C - NK92 Arachidonic acid rep4, TMTpro-131 C - NKL Control rep3, TMTpro-132 C - NKL Control rep4, TMTpro-133 C - NKL Arachidonic acid rep3, TMTpro-134 C - NKL Arachidonic acid rep4, TMTpro-135 N - reference). TMT labelling was performed according to the manufacturer´s instructions.

Following sample mixing, the volume was reduced by half using evaporation to remove acetonitrile. Trifluoroacetic acid was added to a final concentration of 0.5%. TMT-mixes were purified using solid-phase extraction on C18 Sep-Pak, Vac-1 cc-100 mg columns (Waters, Ireland) according to the manufacturer’s instructions. The eluate was split into approximately 10% for downstream proteome analysis and approximately 90% for subsequent phospho enrichment. Both aliquots were evaporated to dryness.

Phosphorylated peptides were enriched using the High-SelectTM Fe-NTA Phosphopeptide Enrichment Kit (#A32992, Thermo Fisher Scientific) according to the manufacturer’s instructions. Both phospho- and proteome aliquots were subsequently fractionated using the Pierce™ High pH Reversed-Phase Peptide Fractionation Kit (#84868, Thermo Fisher Scientific). The entire procedure resulted in the generation of 36 MS samples: 9 protein and 9 phospho fractions per TMT mix. Dried fractionated peptides were resuspended in 0.1% formic acid (FA) prior to LC-MS analysis. For proteome fractions, 5 µl (approximately 500 ng of peptides, assuming 50% sample loss a well as equal sample distribution among fractions for reverse phase fractionation) were injected. For phospho fractions, 10 µl were used for injection.

### Mass spectrometry

Peptides were analyzed by liquid chromatography-tandem mass spectrometry (LC/MS2) on an Exploris 480 instrument connected to an Ultimate 3000 rapid separation liquid chromatography (RSLC) nano instrument and a nanospray flex ion source (all Thermo Fisher Scientific). Peptide separation used a reverse-phase high-performance liquid chromatography (HPLC) column (75 μm by 42 cm) packed in-house with C18 resin (2.4 μm; Dr. Maisch HPLC GmbH, Ammerbuch, Germany). The peptides were first loaded onto a C18 precolumn (preconcentration set-up) and then eluted in the backflush mode using a gradient from 98% solvent A (0.15% formic acid) and 2% solvent B (99.85% acetonitrile and 0.15% formic acid) to 25% solvent B over 48 min, followed by an additional ramp to 35% of solvent B in 20 min. The flow rate was set to 300 nL/min. Data were acquired in a data-dependent mode (DDA) using one high-resolution MS scan at a resolution of 60,000 (m/z 200) with a scan range of 320–1650 m/z, followed by DDA scans limited to a2 sec cycle time, with the first mass set to 199 m/z at a resolution of 45,000. Detailed settings are uploaded with the mass spectrometric raw data to the ProteomeXchange Consortium with dataset identifier: PXD049958, via the MassIVE partner repository (https://massive.ucsd.edu/; MassIVE ID: MSV000094127; doi:10.25345/C50G3H87N).

Peptide spectrum matching was performed using MaxQuant (version 2.4.11.0) against the Human UniProt database (20429 entries, November 2023) with TMT quantification and reporter ion distribution correction (uploaded in the repository). Output was filtered to a 1% false discovery rate on the peptide and protein levels, both, tryptic cleavage following K*, R*, as well as a maximum of 2 missed cleavage. Cysteine carbamidomethylation was included as a fixed modification, while methionine oxidation, asparagine and glutamine deamidation, as well as serine, threonine and tyrosine phosphorylation were set as variable modifications. The full list of settings may be found in the “mqpar.xmL” file uploaded to the ProteomeXchange repository.

### MS / phosphoproteome data analysis

Downstream differential expression analysis was performed in R using the in-house package autonomics, included in the BioConductor collection of R packages (https://doi.org/doi:10.18129/B9.bioc.autonomics). Source code is available at https://gitlab.uni-marburg.de/fb20/ag-graumann/software/autonomics. Autonomics provides an intuitive integrated environment for omic data analysis with an emphasis on a unified interface to linear modeling approaches including coding systems and modeling engines. The implemented functionality defaults to limma as the modeling engine, which was used here. Full analysis code with package versions and settings may be found included in the upload to ProteomeXchange. In brief, MaxQuant output tables “proteinGroups.txt” and “Phospho (STY)Sites.txt” were imported into the R environment. Corrected reporter intensities were normalized by the reference channel intensity, and intensities of phospho-peptides subsequently normalized to their respective protein levels prior to statistical analysis using linear modeling. All data analysis scripts and statistical output is uploaded to the repository. Functional annotation of AA-regulated phosphoproteins was carried out by overrepresentation analysis using the ConsensusPathDB (CPDB) database [44, 45].

### Immunoblots

Immunoblotting was performed according to standard protocols. Treated NK92 cells were centrifuged at 300 g for 5 min, washed with ice-cold PBS and lysed in RIPA lysis buffer (10 mm Tris–HCl pH 7.5, 150 mm NaCl, 1% v/v NP40, 1% w/v sodium deoxycholate, 1 mm EDTA) plus protease inhibitor cocktail (1:1000; Sigma-Aldrich) and phosphatase inhibitor mix (50 mm β-glycerophosphate, 1 mm sodium orthovanadate, 10 mm sodium fluoride and 5 mm sodium pyrophosphate). Equal amounts of proteins were loaded on SDS-polyacrylamide electrophoresis (PAGE) gel and then blotted to polyvinylidine difluoride PVDF membranes (0.45 μm; Carl Roth) by wet transfer. Blots were blocked with 3% BSA or 5% milk in PBS with 0.1% Tween 20 for 1 h at room temperature, incubated with primary antibodies at 4 °C overnight, and followed by 1 h room temperature incubation with HRP-conjugated secondary antibody. The following antibodies were used: p-STAT1 (T701; #612132; BD Bioscience), STAT1 (#9172, Cell Signaling), pERK (T202/Y204; #4370, Cell Signaling, Frankfurt, Germany), ERK (#9107, Cell Signaling), pVASP(Ser293; #3114, Cell Signaling), VASP (Cell signaling, #3112S), pGSK3α(Ser21; #9331, Cell Signaling), and GSK3α (Cell signaling, #5676S). Chemiluminescent and quantification were carried out using the ChemiDoc MP system and image lab software version 5 (Bio-Rad). Phosphoform signals were normalized against the respective protein signals.

### RNA sequencing (RNA-Seq)

RNA was isolated from primary human NK cells as above and libraries were constructed using the ‘Lexogen Quantseq 3′ mRNA-Seq Library Prep Kit FWD for Illumina’ (Lexogen, Vienna, Austria) in combination with the ‘Lexogen UMI Second Strand Synthesis Module for QuantSeq FWD (Illumina, Read 1)’, according to the manufacturer’s instructions. Quality of libraries was controlled on a Bioanalyzer 2100 using the Agilent High Sensitivity DNA Kit (Agilent, Waldbronn, Germany). Pooled sequencing libraries were quantified and sequenced on the Illumina NextSeq550 platform (San Diego, CA, USA) with 75 base single reads.

Data were aligned to the human genome retrieved from Ensembl 108 using star (version STAR_2.7.10a). Gene read counts were determined within merged exons of protein-coding transcripts or within merged exons of all transcripts (for noncoding genes) and calculated as CPM (counts per million). RNA-Seq data was deposited at EBI ArrayExpress (accession number E-MTAB-13971).

Differential gene expression (RNA-Seq data) was calculated with edgeR [46] and DESeq2 [47] using standard parameters and paired analysis (on donors). Pathway analysis of differentially expressed genes was performed using the online tool of the Database for Annotation, Visualization and Integrated Discovery (DAVID; https://david.ncifcrf.gov) [48].

TANK were purified from ascites using density gradient centrifugation and NK cells were then isolated using the NK Cell Isolation Kit 130-092-657 (Miltenyi Biotec, Bergisch Gladbach, Germany). RNA was isolated using the phenol-chloroform-extraction and DNA contamination was removed with the DNA-free™ DNA Removal Kit (Invitrogen) according to the manufacturer’s instructions. The quality of the RNA was verified using the Experion RNA StdSens Analysis Kit or using the Qubit fluorometer (Thermo Fisher Scientific). Next, RNA-libraries were generated from total RNA by using the TruSeq Stranded mRNA Library kit according to the manufacturer’s instructions. The sequencing was conducted on an Illumina HiSeq 1500 device. Alignment was performed as for NK cells. Read counts were quantified in exonic regions of protein-coding transcripts and normalized to transcripts per million reads (TPM). TANK data was deposited under accession number E-MTAB-14,053.

To compare NK cells from PMBCs with those from ascites s a correction for contaminating tumor RNA was necessary. Tumor cell data from a previously published study [40] was retrieved from EBI ArrayExpress (identifiers E-MTAB-10611, E-MTAB-4162 and E-MTAB-5498) and processed identically to the TANK data.

To compensate for batch effects and differences in RNA-Seq library preparation and quantification methods (QuantSeq and Counts Per million for NK-cell samples, Illumina TruSeq mRNA stranded and Transcripts per Million for Tumor/TANK samples), we applied Trimmed Mean of M-values [49]. Genes below a minimum expression threshold (TMM_NK > 0 or TMM_TANK > 0) were excluded from further analyses. Furthermore, due to the observed contamination of TANKs with tumor cells, we refined the gene list by filtering for genes meeting one of the following criteria (i) a > 2-fold higher expression in NK compared to TANK [median(TMM_NK) > (median(TMM_TANK) + 1], or (ii) negligible expression in tumor cells [max(TMM_tumor) < 0)] or (iii) a markedly lower expression in tumor cells compered to TANK [max(TMM_tumor) < min(TMM_TANK) -1]. Subsequently, we applied a gene-wise Student’s t test followed by Benjamini Hochberg correction to evaluate significance. For a description of significance levels, refer to the section on statistical methods.

### Quantitative reverse transcriptase polymerase chain rection (RT-qPCR)

2 × 10^5^ primary human NK cells were incubated with solvent, AA, IL-2 (200 IU/mL), IL-2 plus AA or ascites (50%) for 3 h. Total RNA was extracted using the NucleoSpin RNA II kit (#740955.250; Macherey-Nagel, Düren, Germany). cDNA was prepared from 250 ng of total RNA with the iScript™ cDNA Synthesis Kit (BioRad, Feldkirchen, Germany) according to the manufacturer’s protocol. Raw data were assessed using the Cy0 method and RPL27 for normalization. The following primer pairs were used:

TNF-for: CAGCCTCTTCTCCTTCCTGAT;

TNF-rev: GCCAGAGGGCTGATTAGAGA;

IFNG-for; GAGTGTGGAGACCATCAAGGA;

IFNG-rev: TGGACATTCAAGTCAGTTACCGAA;

RPL27-for: AAAGCTGTCATCGTGAAGAAC;

RPL27-rev: GCTGTCACTTTGCGGGGGTAG.

### Reactive oxygen species (ROS) assay

Primary NK or NK92 cells were seeded in 24-well plates at 10^6^ cells/mL. A total ROS/superoxide detection kit (#ENZ-51010; Enzo Life Sciences, Lörrach, Germany) was used to measure total ROS concentration according to the manufacturer’s protocol. In brief, NK92 cells were preincubated with the ROS scavenger N-acetylcysteine (NAC; 5 mM) for 30 min prior to treatment with solvent or 50 µM AA for 1 h. prior to staining with ROS detection reagents. NAC-treated samples served as negative controls. Cells were washed once with the assay buffer and ROS was measured on a Guava^®^ easyCyte Flow Cytometer (Cytek^®^ Biosciences, CA, USA).

### ELISA

Supernatants from 2 × 10^5^ primary NK cells were treated with solvent, AA, IL-2 (200 IU/mL) or IL-2 plus AA and collected 48 h post-treatment. IFNγ concentrations were measured using the DuoSet™ ELISA (#DY285B-05, R&D Systems/Biotechne, Wiesbaden, Germany) according to the manufacturer’s instructions.

### Surface receptor expression

5 × 10^5^ primary human NK cells were cultured with solvent, AA, IL-2 (10 ng/mL) plus IL-15 (10ng/mL) or AA plus IL-2 and IL-15 for 24 h, washed with PBS and stained with CD3-V500 [#561416; Becton, Dickinson and Company (BD)], CD16-APC-Cy7 (#302018; BioLegend, San Diego, CA, USA), CD56-PerCP-Cy5.5 (#362506; BioLegend), NKG2D-FITC (#320820; BioLegend, supplier Biozol, Eching, Germany), NKp44-AF647 (#325112; BioLegend), NKp30-BV421 (#563385; BD), NKp46-PE (#331908; BioLegend) and CD69 (clone FN50, BioLegend). The following corresponding IgG controls were used: IgG1K-V500 (#560787; BD), IgG1K-APC-Cy7 (#400128; Biolegend), IgG1K-PerCP-Cy5.5 (#400150; Biolegend), IgG1K-FITC (#400108; Biolegend), IgG1K-AF647 (#400130; Biolegend), IgG1K-BV421 (#562438; BD) and IgG1K-PE (#400114; Biolegend). Cells were incubated at 4 °C for 15 min and washed twice with ice cold FACS staining buffer (PBS with 1% FBS and 0.5 mM EDTA). Surface expression of receptors was analyzed by flow cytometry (FACS Canto II, BD Biosciences) using FlowJo software. To determine the mean fluorescence intensity (MFI), the background geometric mean of the isotype was subtracted from the geometric mean of the specific staining of each sample.

### Human phosphokinase array

To determine the relative phosphorylation of 37 kinases, a phospho-kinase array kit (#ARY003C; R&D Systems) was used according to the manufacturer´s protocol. In brief, 2 × 10^6^ NK92 cells were stimulated with IL-2 (200 U/mL) and IL-15 (10 ng/mL) and incubated overnight. In parallel, a 12-well plate was coated with purified anti-human NKG2D (#320802; Biolegend) and its respective IgG control (#400102; Biolegend) at a concentration of 10 µg/mL and kept at 4˚C overnight. The next day, NK92 cells were pre-incubated with AA or with solvent for 15 min before being treated with α-NKG2D and isotype coated well for an additional 15 min. After incubation, cells were lysed in lysis buffer, followed by incubation on a rocker at 4˚C for 30 min and centrifuged at 14000x g for 5 min. Afterwards, the protein concentration in the resulting supernatants was then measured using the BCA assay (#23227, Thermo Fischer Scientific). Membranes (A and B) were blocked in array buffer 1 for an hr at room temperature. Subsequently, 350 µg of protein in each sample was incubated overnight at 4˚C, followed by washing with wash buffer and incubation with the appropriate detection antibody cocktail (DAC-A and DAC-B) for 2 h at room temperature. Later, membranes were then incubated with streptavidin-HRP for 30 min and after thorough washing; blots were developed using 1 mL chemi-reagent mix for each pair of membrane and chemiluminescent detection system. The pixel density of each spot was calculated by using ImageJ software with the protein array analyzer plugin.

### MICA immobilization

Recombinant MICA protein (#E-PDMH100040; Biomol, Hamburg, Germany; 5 µg /mL) was immobilized on 48-well tissue culture plates for 24 h at 4 °C. Wells were gently rinsed with phosphate-buffered saline (PBS), blocked with 3% bovine serum albumin in PBS for 1 h at room temperature and rinsed again with PBS. 5 × 10^5^ NK92 cells pretreated with AA or solvent for 15 min were added to the coated or uncoated wells and incubated for 20 min at 37 °C. Cells were collected by centrifugation and cell extracts were analyzed by immunoblotting.

### Proliferation assay

Proliferation of NK92 cells was monitored using CellTrace™ CFSE Cell Proliferation Kit (#C34554; Fischer) according to the manufacture’s protocol. Briefly, 1 mL of cell suspension (10^6^ cells/mL) were stained with 1 µL of CellTrace™ for 20 min at 37 °C. Next, 5 mL of RPMI medium containing 10% FBS were added to the cell suspension. Labeled cells were harvested by centrifugation, resuspended in RPMI medium containing 10% FBS plus IL-2 (10 U/mL) and and seeded in a U-shaped 96-well plate. 5 × 10^5^ cells were treated with solvent, IL-2 (200 IU/mL) or IL-2 plus AA dissolved in 96% ethanol, and proliferation of cells was assayed after 3 days by flow cytometry (FACSCanto II, BD Biosciences, Heidelberg, Germany). Acquired data were analyzed by FlowJo software (Becton, Dickinson & Company, Heidelberg, Germany).

### NK cells cytotoxic assay with K562 target cells

5 × 10^5^ K562 cells were stained with 5 µM CellTracker™ Violet BMQC (Thermo Fischer Scientific) for 45 min at 37 °C following the instruction of the manufacturer.10^6^ NK92 cells were stimulated with IL-2 (200 U/mL) plus IL-15 (10 ng/mL) overnight, then incubated with AA or with solvent for 30 min and finally added to the stained K562 cells at an effector: target ratio of 4:1 in a final volume of 200 µl. In parallel, 10^5^ K562 cells were incubated with AA or solvent to determine potential AA-induced toxicity on K562 cells. After 3 h of incubation at 37 °C, cells were collected by centrifugation at 300 × g for 5 min and stained with by propidium iodide (PI) staining (Cay14289-10, Cayman Chemical, supplier Biomol) at a final concentration of 1 µg/mL. Cell viability was measured by flow cytometry (FACSCanto II, BD Biosciences) and analyzed by the FlowJo application.

### NK cells cytotoxic assay with OVCAR-8 target cells

10^6^ NK92 cells were stimulated with IL-2 (200 U/mL) and IL-15 (10 ng/mL) and were incubated overnight to induce their activation. On the next day, OVCAR-8 parental cells were stained with 5µM CellTracker^™^ violet BMQC (Invitrogen) fluorescent dye in serum-free medium for 45 min at 37˚C. NK92 cells were incubated with AA or with a solvent with 50µM for 30 min and then added to the stained OVCAR-8 cells at the indicated effector: target (E: T) ratio into a round bottom 96-well microplate. In parallel, OVCAR-8 cells were incubated with AA or solvent with the same concentration to determine potential AA-induced toxicity on OVCAR-8 cells. The plate was centrifuged at 100x g for 1 min to bring cells into proximity and incubated for 3 h at 37˚C. Cells were collected and analyzed as described in the preceding paragraph.

### Other statistical analyses

Paired Student’s t-test statistical analysis (two-sided, equal variance) was applied for analyzing comparative western blot, FACS, ROS and RT-qPCR data. Levels of significance are indicated as ****, ***, **, and * for *p* < 0.0001, *p* < 0.001, *p* < 0.01, and *p* < 0.05, respectively.

## Results

### The AA-regulated phosphoproteome of NK cell lines: impact on signal transduction pathways

**Fig. 1:**
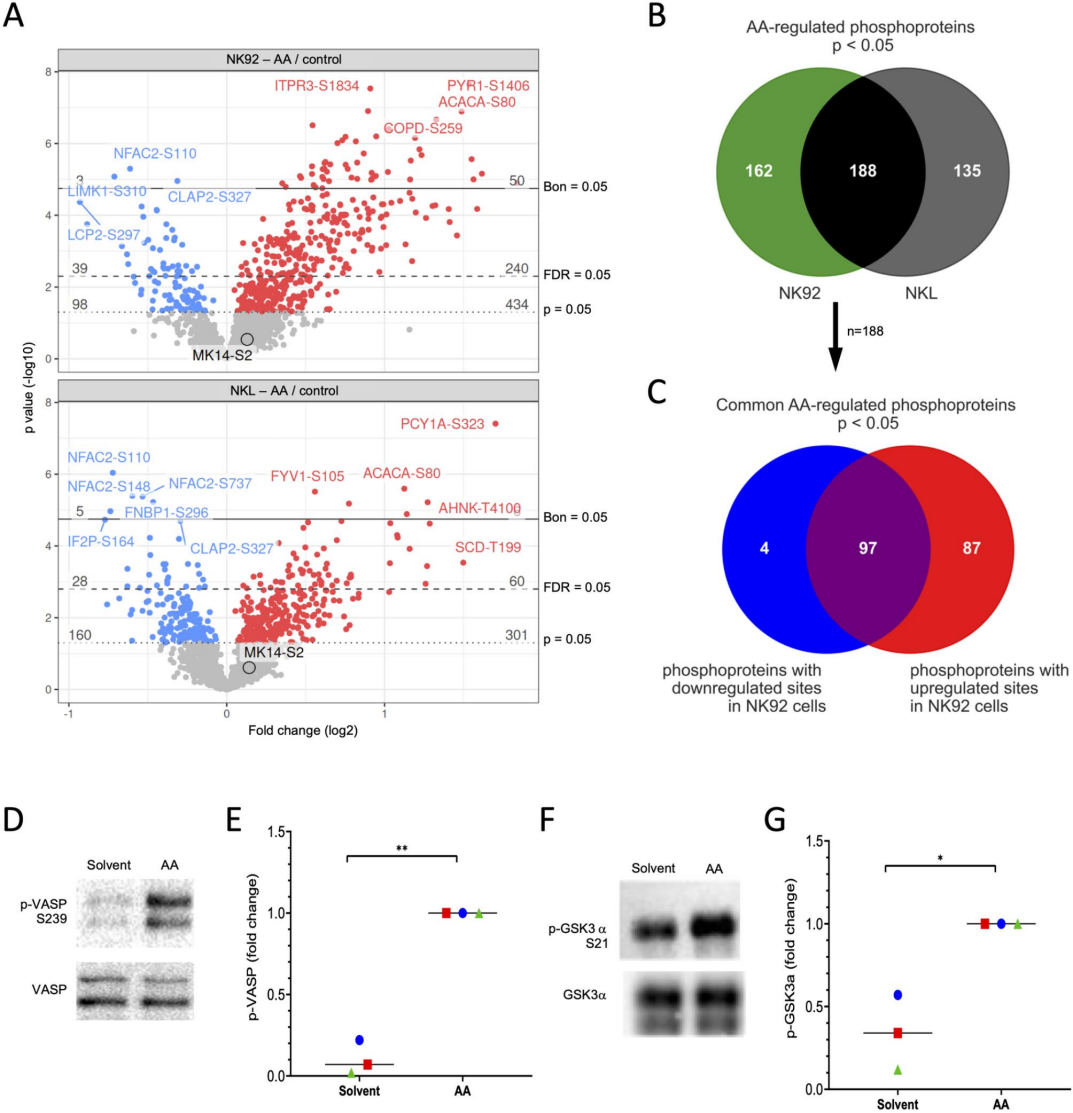
Effect of AA on the phosphoproteome of NK92 and NKL cells. Cells were treated with 50 µM AA or solvent for 15 min following serum starvation for 24 h. **(A)** Volcano plots showing phosphosites regulated by AA in NK92 (top panel) and NKL (bottom panel) cells. Blue: downregulated sites (*p* < 0.05). Red: upregulated sites (*p* < 0.05). Grey: sites not significantly affected. Dashed lines shown the significance thresholds and to the right of them the corresponding numbers of downregulated (blue) and upregulated (red) genes. Bon: Bonferroni correction. The top regulated phosphosites are indicated in both volcano plots. **(B)** Venn diagram showing the overlap of proteins with regulated phosphosites in NK92 and NKL cells for significantly sites (*p* < 0.05; see Fig. S1 for FDR < 0.05). **(C)** Venn diagram depicting the overlap of common proteins with upregulated and down-regulated phosphosites in NK92 cells (*n* = 188 in panel B). **(D)** Validation of phosphoproteomic data for VASP by immunoblotting (representative experiment). NK92 cells were treated with 50 µM AA or solvent for 15 min following serum starvation for 24 h. The data confirm the observed phosphorylation at serine 239 (Table S1). **(E)** Quantification of *n* = 3 independent experiments as in panel D). **(F,G)** Validation of phosphoproteomic data for AA-regulated phosphorylation of S21 in GSK3α. **p < 0.01, *p < 0.05 by paired t test

To unravel the AA-regulated signaling pathways in NK cells by MS-based phosphoproteomics, we employed established cell lines. This choice was made because primary NK cells exhibit significant donor variability, requiring a large number of samples to detect statistically meaningful AA-triggered phosphosite modifications (> 2-fold changes in signal intensities in most cases; see data below). Accordingly, we analyzed NK92 cells treated with AA or solvent for 15 min, which revealed *n* = 2,800 phosphorylation sites detectable across all samples and associated with known genes (see Table S1). After individually adjusting for protein group signal, we identified *n* = 434 upregulated phosphorylation sites (*p* < 0.05; *n* = 240 of which had an FDR < 0.05) and 98 downregulated sites (*p* < 0.05; *n* = 39 with FDR < 0.05) across 350 proteins (Fig. 1A, top panel; Fig. 1B and S1; Table S1). To corroborate these findings, we examined a second NK cell line (NKL cells) under identical conditions (Table S2), discovering *n* = 301 upregulated sites (*p* < 0.05; *n* = 60 with FDR < 0.05) and *n* = 160 downregulated sites (*p* < 0.05; *n* = 28 with FDR < 0.05) across 323 proteins (Fig. 1A, bottom panel and Fig. 1B; Table S2). There was a significant overlap in the AA-modulated phosphoproteins between the two cell lines, with 188 proteins being common, representing 53.7% and 58.2% of the regulated phosphoproteins in NK92 and NKL cells, respectively. Notably, a considerable portion (*n* = 97 out of *n* = 188 proteins, or 51.6%) exhibited both upregulated and downregulated phosphorylation sites in NK92 cells (Fig. 1C). These findings suggest that the majority of the identified phosphorylation sites are broadly relevant rather than specific to a particular cell line. Given the more extensive dataset of AA-regulated phosphorylation sites in NK92 cells, our subsequent analyses predominantly focused on this cell line. Immunoblotting confirmed the phosphoproteomics data for two proteins (Table S1) for which the corresponding phosphosite-specific antibodies were available, i.e., S239 in VASP (Fig. 1D and E) and S21 in GSK3α Fig. 1F and G).

**Fig. 2:**
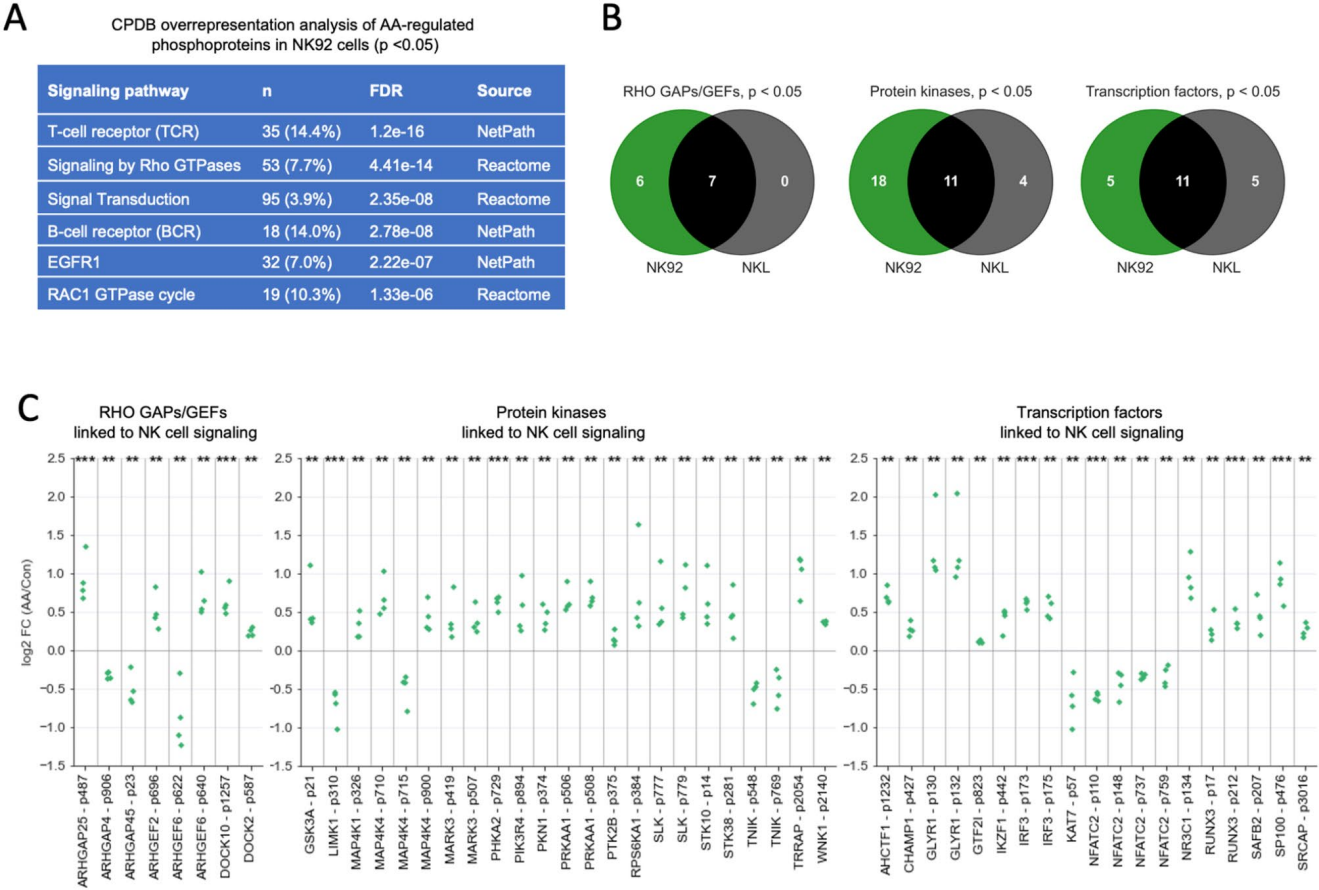
Analysis of proteins with AA-regulated phosphosites. **(A)** Functional annotation by CPDB overrepresentation analysis of AA-regulated phosphoproteins in NK92 cells (*p* < 0.05; *n* = 162 + 188 in Fig. 1B). The table shows the top non-redundant terms. n: number of proteins associated with the respective term. **(B)** Functional annotation of AA-regulated phosphoproteins according to their molecular function based on the Human Protein Atlas database. **(C)** AA-mediated regulation in NK92 cells of phosphosites in RHO GAPs/GEFs, protein kinases and transcription factors linked to NK cell activation in the Genecards database. The plots show the log2 FC (AA/solvent) for *n* = 4 replicates (green dots). Upregulated sites (log2 FC > 0) and downregulated sites (log2 FC < 0) are separated by a horizontal line. Significance was tested by paired t test: ***FDR < 0.001; **FDR < 0.01

CPDB overrepresentation analysis of AA-modulated phosphoproteins in NK92 cells (*n* = 350; depicted in green and black in Fig. 1B) revealed key signal transduction pathways as predominant terms, including receptor-mediated and RHO/RAC1 signaling (Fig. 2A). This finding is further supported by the categorization of AA-affected phosphoproteins based on their molecular function, highlighting the involvement of 13 RHO GAPs/GEFs, 29 protein kinases and 16 transcription factors, which also showed a strong overlap with the regulated phosphoproteins in NKL cells (Fig. 2B). Intriguingly, many of these proteins have been linked to NK cell activation in the GeneCards database [50, 51]. AA-mediated regulation of phosphosites in these proteins in NK92 cells (FDR < 0.05) is illustrated in detail for 4 biological replicates in Fig. 2C. These encompass multiple proteins with reported roles in NK cell signal transduction and biological functions, including.

(i) Regulators and targets of RHO/RAC1 pathways [52, 53], i.e. guanine exchange factors (ARHGEF and DOCK family members), GTPase activating proteins (ARHGAPs) and the RHO kinase target LIM kinase 1 (LIMK1),
(ii) Protein kinases glycogen synthase kinase 3 (GSK3) [54, 55] and PKA [56, 57], as well as.
(iii) The transcription factors IKFZ1/IKAROS [58, 59], NFAT [60, 61] and RUNX3 [58, 62–64].

Collectively, our phosphoproteomic analysis suggests a profound impact of AA on signaling pathways crucial for the regulation of NK cell functions.

### The AA-regulated transcriptome of NK cells: impact on ROS detoxification and receptor-mediated signaling

**Fig. 3:**
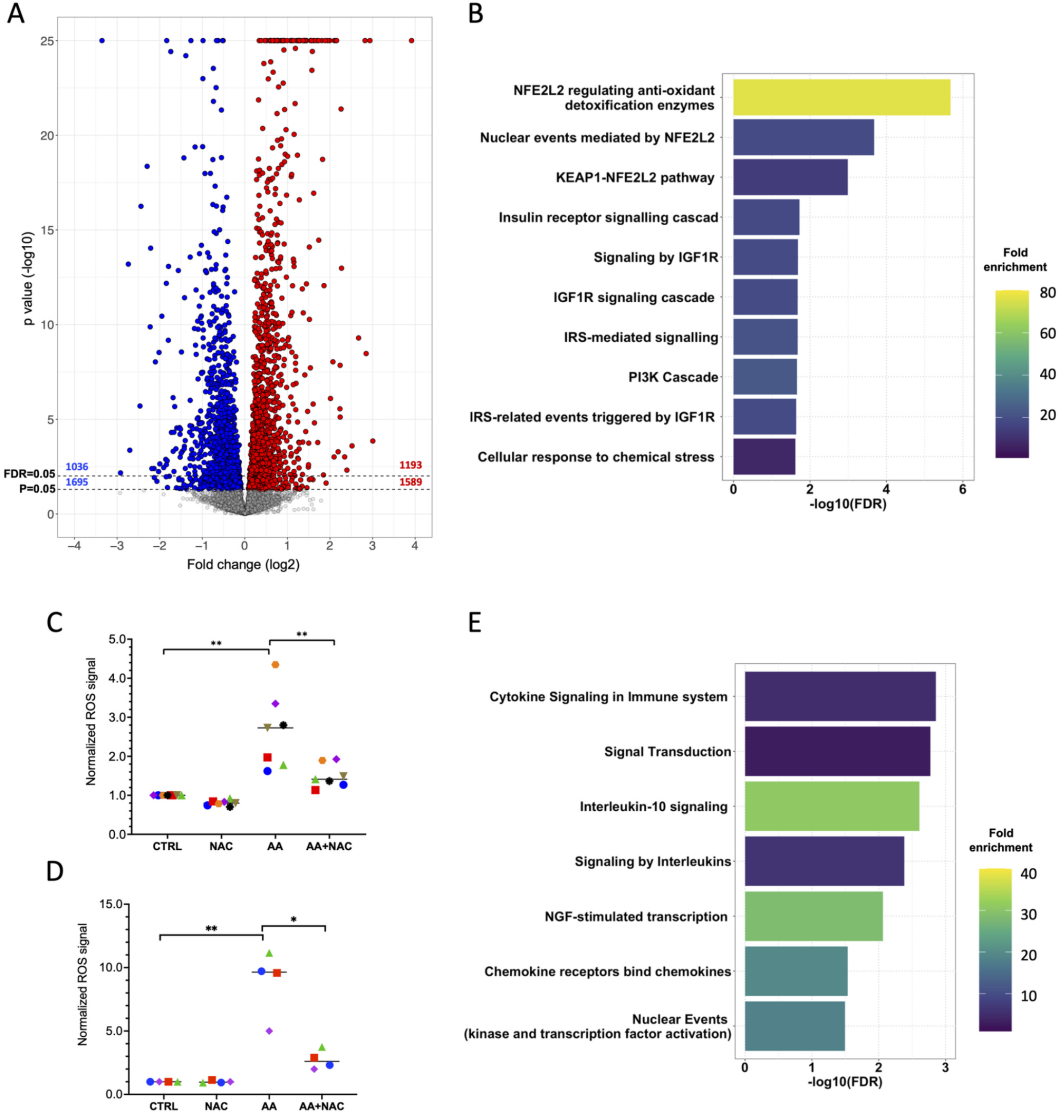
AA induces ROS detoxification genes in primary NK cells. Primary NK cells were exposed to AA or solvent for 3.5 h and analyzed by RNA-Seq. **(A)** Volcano plot showing genes regulated by AA. Blue: downregulated genes (*p* < 0.05); red: upregulated genes (*p* < 0.05). Grey: genes not significantly affected. Dashed lines shown the significance thresholds and above the corresponding numbers of downregulated (blue) and upregulated (red) genes. **(B)** Functional annotation of AA-induced genes using the DAVID online tool [48]. **(C**,**D)** Primary NK cells (C) and NK92 cells (D) were treated with AA or solvent (CTRL) for 1 h and intracellular ROS levels were determined by flow cytometry using a total ROS detection kit (see Fig. S2 for a representative histogram). The ROS scavenger NAC was included to verify the induction of ROS by AA (30 min pretreatment). **p < 0.01; *p < 0.05 by paired t test for *n* = 7 biological replicates in panel C and *n* = 4 in panel D. **(E)** Functional annotation of AA-repressed genes using the DAVID

To explore the intricacies of the signal transduction network modulated by AA in NK cells, we conducted RNA-Seq analysis on primary NK cells from healthy donors treated with 50 µM AA or a solvent control for 3.5 h (Table S3). The volcano plot resulting from the differential expression analysis and presented in Fig. 3A, indicates that *n* = 1,589 genes were significantly upregulated (*p* < 0.05; of these, 1,193 had an FDR < 0.05), while 1,695 genes were significantly downregulated (*p* < 0.05; with 1,036 exhibiting an FDR < 0.05).

Genes induced by AA by ≥ 2-fold were functionally annotated using the DAVID Bioinformatics Resource [48]. This analysis identified NFE2L2-mediated regulation of reactive oxygen species (ROS) as the primary mechanism affected, as shown in Fig. 3B. AA-induced downstream targets of this pathway identified by the PANTHER Classification System [65, 66] include *GSR* (glutathione reductase), *PRDX1* (peroxiredoxin-1), PRDX6 (peroxiredoxin-6), *SOD1* (superoxide dismutase), *TXN* (thioredoxin), and *TXNRD1* (thioredoxin reductase 1) (expression data in Table S3).

The transcription factor NFE2L2, which is inducible by reactive oxygen species (ROS), plays a pivotal role in cellular defense mechanisms against ROS [67]. It achieves this by enhancing the expression of detoxification enzymes, such as those identified in AA-treated cells (refer to Table S3). This pattern suggests that the upregulation of these genes by AA is mediated through the induction of ROS. To investigate this hypothesis, we treated primary NK cells and NK92 cells with AA or a solvent control for 1 h and subsequently assessed intracellular ROS levels using flow cytometry (see Fig. S2). The results depicted in Fig. 3C and D demonstrate an approximately 3-fold and 10-fold increase of ROS in primary NK and NK92 cells, respectively, following AA treatment compared to the solvent control. Notably, this elevation in ROS was significantly reduced by > 50% upon treatment with the ROS scavenger NAC.

The analysis of genes downregulated by AA highlighted terms associated with cytokine signaling as the predominant findings (Fig. 3E). Among these are *CX3CR1* and *CXCR3* (Table S3), which play pivotal roles in the homing of NK cells to peripheral tissues, including tumor sites [68]. Taken together, these insights underline the multi-faceted impact of AA on NK cells, notably influencing both ROS regulation and cytokine/chemokine signal transmission essential for their functional performance.

### Impairment of cytokine signaling in NK cells by AA through inhibition of STAT1 phosphorylation

**Fig. 4:**
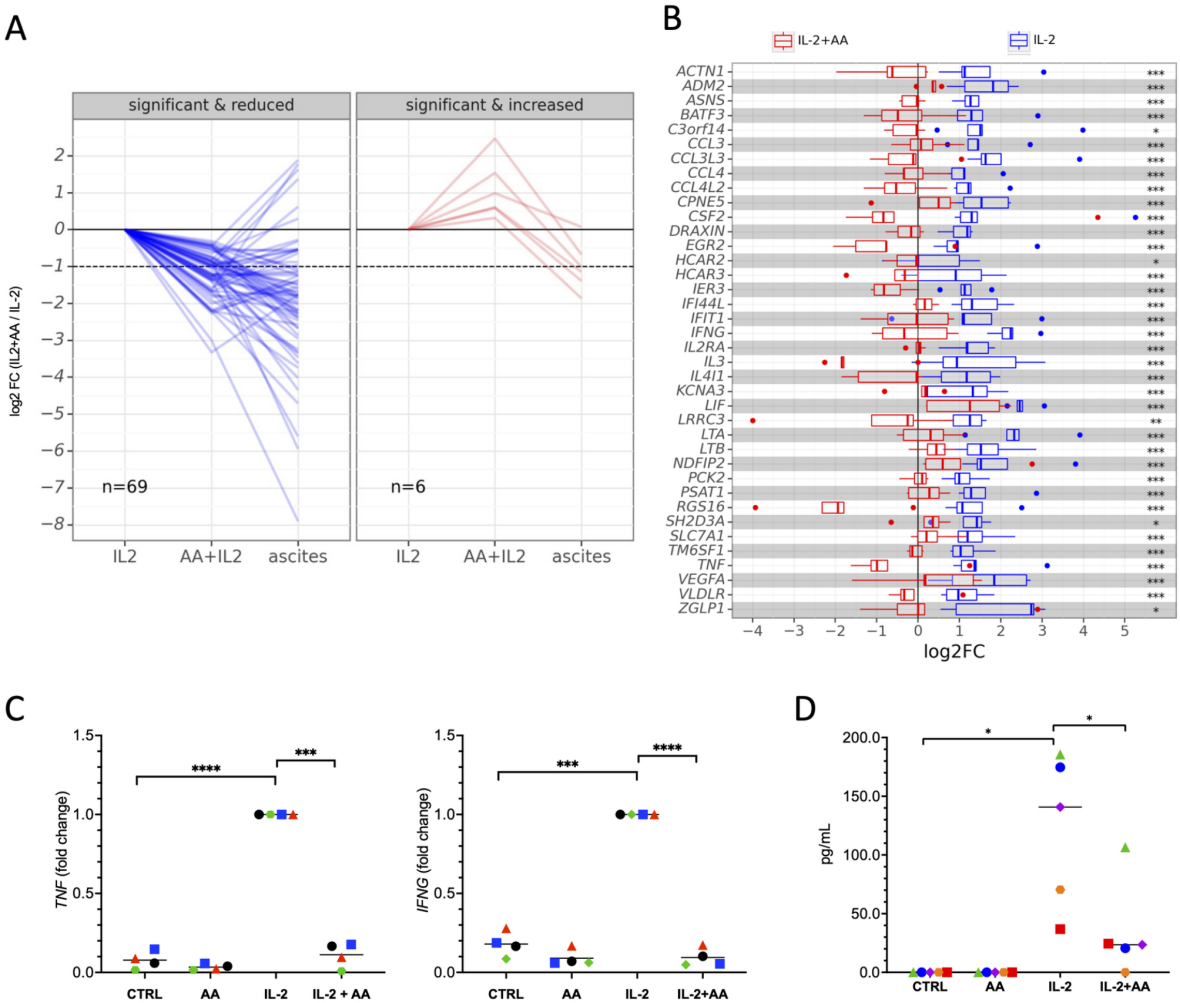
AA inhibits IL-2 induced genes in primary NK cells. Primary NK cells were exposed to IL-2 (200 IU/mL) or IL-2 plus AA for 3.5 h and analyzed by RNA-Seq. **(A)** Genes showing reduced IL-2-mediated induction in the presence of AA. Each line represents the median of *n* = 5 replicates expression of one gene under different conditions (normalized to IL-2). NK cells incubated with 50% ascites for 3.5 h were included for comparison. Left panel: significant (FDR < 0.05) repression by AA; right panel: significant upregulation by AA. **(B)** Expression levels of the IL-2-induced genes significantly repressed by AA (FDR < 0.05) for *n* = 5 biological replicates (NK cell donors). The plot shows the median (line), upper and lower quartiles (box), range (whiskers) and outliers (dots). **(C)** Verification of RNA-Seq data by RT-qPCR for *TNF* and *INFG* (*n* = 4 biological replicates). Data were normalized to 1.0 for IL-2 treatment. **(D)** AA inhibits the IL-2-induced secretion of IFNy by primary NK cells. IFNy levels were determined in culture supernatants 48 h post-stimulation by ELISA (*n* = 5 biological replicates). ****p < 0.0001; ***p < 0.001 1; *p < 0.05 by paired t test

Building on the observations in Fig. 3B-E, we explored the effect of AA on the transcriptional response to IL-2, a cytokine critically involved in promoting NK cell proliferation and cytotoxicity [69]. To investigate this, we exposed primary NK cells to IL-2 alone or in combination with AA for 3 h, followed by analysis using RNA sequencing (RNA-Seq; Table S4). Figure 4A highlights the impact of AA on IL-2-induced gene expression, demonstrating repression of *n* = 69 out of *n* = 75 IL-2-responsive genes by AA in the left panel (FDR < 0.05), and an amplification of IL-2-induced expression for *n* = 6 genes in the right panel. The vast majority of AA-repressed genes (63 out of 69) were also downregulated when NK cells were incubated with 50% ascites (Fig. 4A). However, the patterns of AA-induced and ascites-regulated gene expression diverged, underscoring the potential significance of AA-mediated suppression of IL-2 induced genes. The specifics of AA-mediated suppression are depicted in Fig. 4B for samples from *n* = 5 NK cell donors.

Validation of the RNA-Seq data was achieved through RT-qPCR, confirming the suppression of IL-2-induced *TNF* and *INFG* expression (Fig. 4C). Consistent with these results, functional annotation of the IL-2 target genes repressed by AA (*n* = 69 in Fig. 3A) pinpointed cytokine signaling as the most affected pathways, notably including IL-2 signaling itself (refer to Fig. S3). This conclusion is supported by the observation that AA inhibited the IL-2-induced secretion of INFγ by primary NK cells (Fig. 4D).

**Fig. 5:**
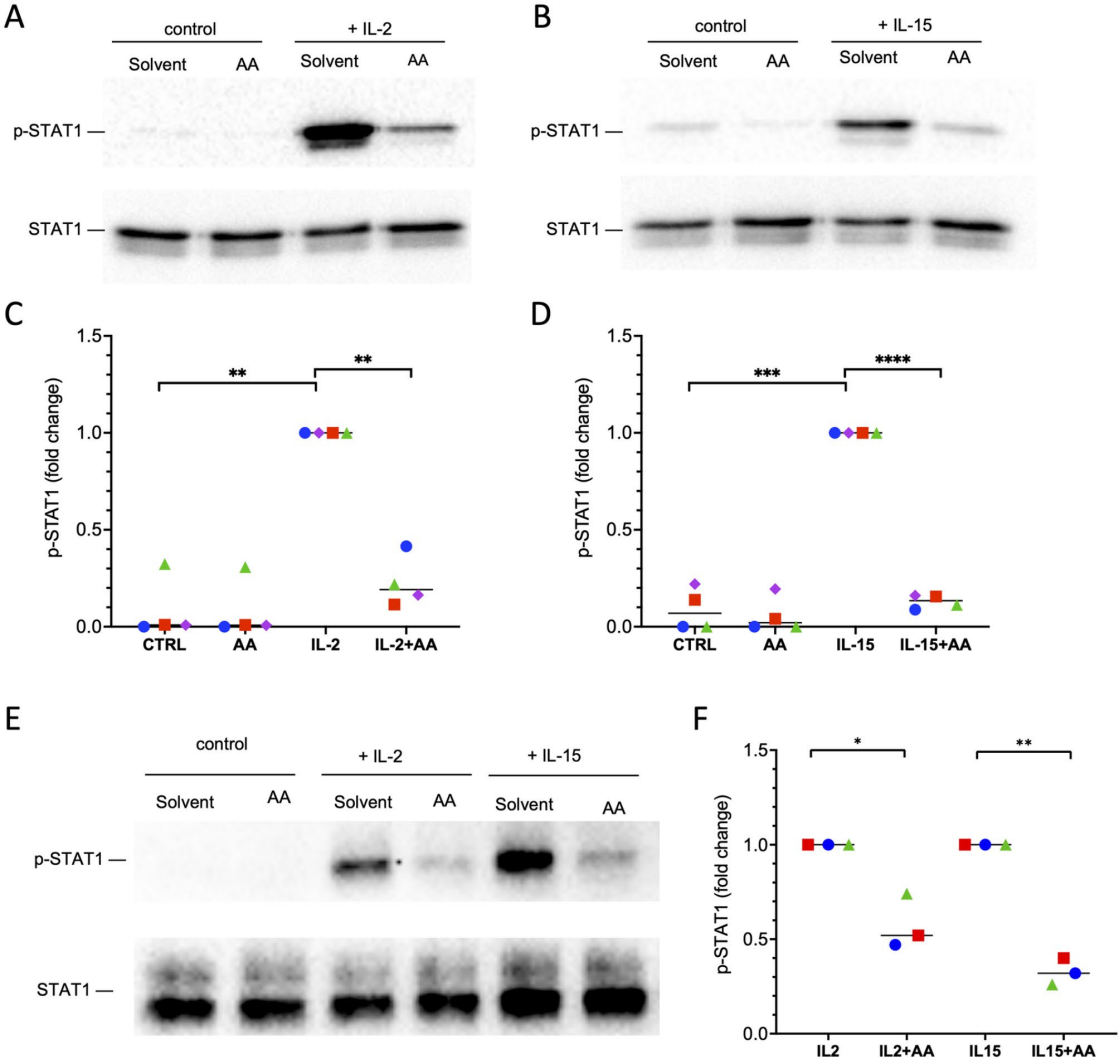
AA impairs cytokine-mediated activation of STAT1 phosphorylation in NK92 and primary NK cells. **(A**,**B)** Representative immunoblots showing the effect of AA on IL-2 and IL-15-induced STAT1 phosphorylation in NK92 cells. Cells were treated with AA 50 µM or solvent for 15 min before stimulation with IL-2 (200 IU/mL; panel A) of IL-15 (20 ng/mL; panel B) for 30 min. Blots were probed with pSTAT1- and STAT1-specific antibodies. **(C**,**D)** Quantification of *n* = 4 replicates of experiments analyzing the effect of AA on IL-2 and IL-15-induced STAT1 phosphorylation as in panels A and B. **(E)** Representative immunoblot showing the effect of AA on IL-2 and IL-15-induced STAT1 phosphorylation in primary NK cells (conditions as in panels A and B). **(F)** Quantification of *n* = 3 biological replicates (donors) of experiments as in panel E. ****p < 0.0001; ***p < 0.001; **p < 0.01; *p < 0.05 by paired t test

We next investigated at which point in the signaling network AA may inhibit cytokine signaling. To this end, we analyzed the effect of AA on cytokine-induced STAT1 phosphorylation. As shown by immunoblotting phosphorylation STAT1 at T-701 was > 10-fold induced in NK92 cells after a 30-minute stimulation with IL-2 (Fig. 5A, C) or IL-15 (Fig. 5B, D), which was inhibited by > 80% following preincubation with AA. Very similar results were obtained with primary NK cells. (Fig. 5E, F). These findings clearly demonstrate that AA interferes with a functionally pivotal signaling mechanism in NK cells.

### Inhibition of activating NK cell receptors by AA

**Fig. 6:**
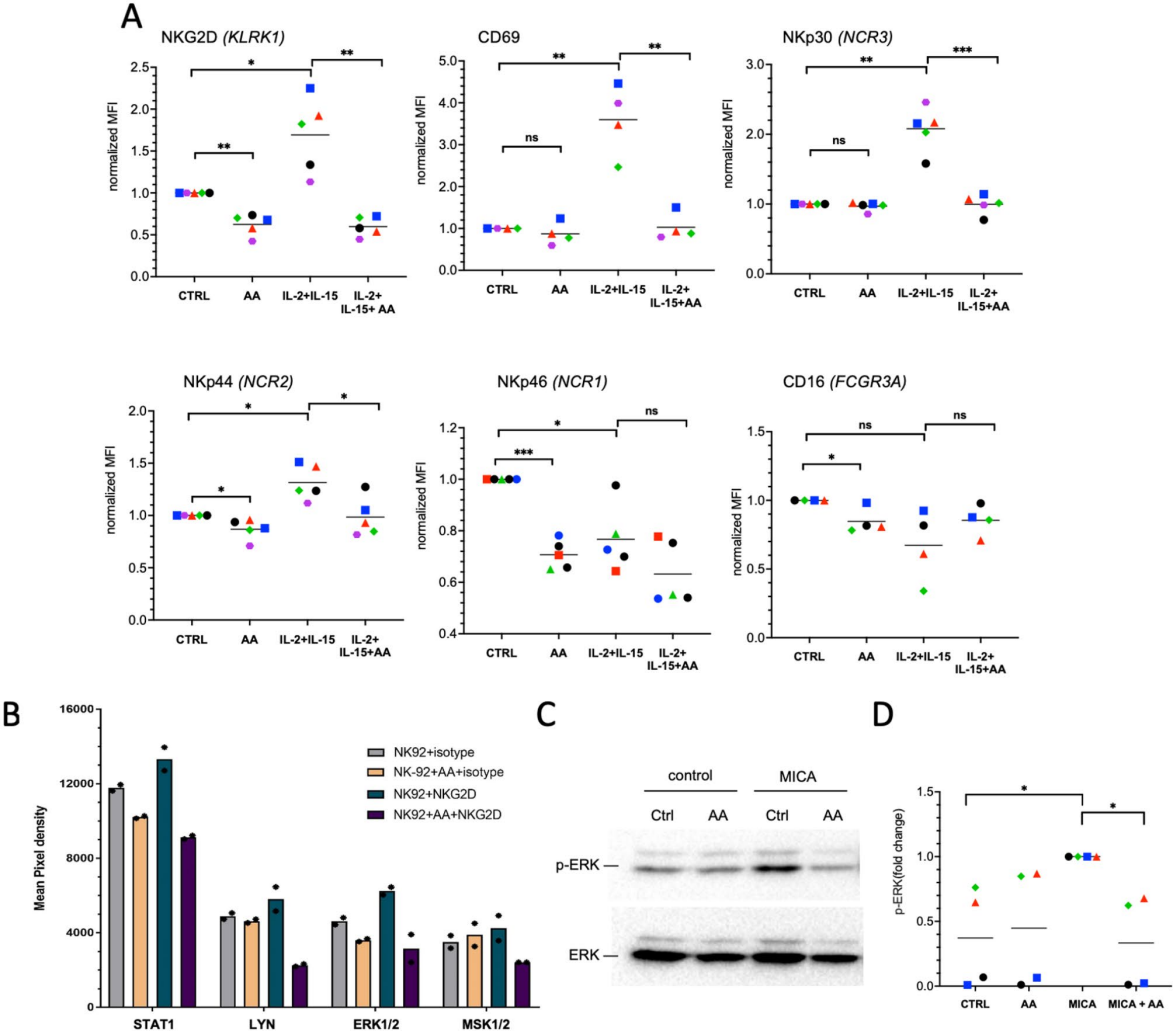
AA inhibits signaling by activating NK cell receptors. **(A)** Flow-cytometric analysis showing the effect of AA on the cytokine-induced surface expression of the indicated NK cell receptors on primary NK cells. Cells were treated with solvent (CTRL), AA, IL-2 plus IL-15 (10 ng/mL each) or AA plus IL-2 and IL-15 for 24 h. The plots show normalized MFI values (CTRL = 1) for *n* = 4–5 biological replicates (different donors). Absolute MFI values and percentage of positive cells are shown in Figs. S4 and S5. **(B)** Effect of AA on the phosphorylation of signaling proteins induced by NKG2D engagement analyzed by the commercial Proteome Profiler Human Phospho-Kinase Array Kit. NK92 were pre-incubated with 50 µM AA or solvent for 15 min followed by incubation with 10 µg/mL α-NKG2D or matching isotype control coated wells for an additional 15 min. Symbols show technical duplicates; bar represent the respective mean. **(C)** Representative immunoblot showing the effect of AA on ERK phosphorylation triggered by immobilized recombinant MICA in NK92 cells. Cells were treated with AA or solvent for 15 min prior to MICA stimulation for 20 min. **(D)** Quantification of *n* = 4 biological replicates of experiments as in panel A. ***p < 0.001; **p < 0.01; *p < 0.05 by paired t test

We next investigated whether AA affects signal transduction by activating NK cell receptors, which are critical effectors of NK-mediated cytotoxicity [70]. Toward this goal, we first analyzed the impact of AA on IL-2/IL-15-induced surface expression of six receptors on primary NK cells. The flow cytometric data depicted in Fig. 6A and S4-S6 show a significant 1.3- to 3.0-fold induction of NKG2D, CD69, NKp30 and NKp44 by combined stimulation with IL-2 and IL-15, while cytokine-treatment did not induce surface expression of NKp46 and CD16. This induction was reversed by AA to baseline levels or lower. Moreover, AA notably reduced the surface expression of NKG2D and NKp46 even in the absence of cytokine stimulation (Fig. 6A, S5 and S6).

Next, we assessed the impact of AA on NKG2D-dependent signal transduction in NK92 cells. NK cell receptors are expressed and NKG2D, NKp44 and NKp30 are inducible by cytokines in this cell line (Fig. S7), indicating that NK92 cells are a suitable experimental model. Accordingly, we investigated the phosphorylation of key signaling proteins in NK92 cells upon NKG2D stimulation with an activating antibody using a membrane-based sandwich immunoassay (Proteome Profiler Human Phospho-Kinase Array Kit). Among the 22 detectable phosphorylation sites, we found that NKG2D engagement-induced phosphorylation of four kinases was repressed by AA. Consistent with the date described above these kinases included STAT1 (Y701) and ERK1/2 (T202/Y204, T185/Y187), along with LYN (Y397) and MSK1/2 (S376/S360) (Fig. 6B). Additionally, the phosphorylation of four kinases downregulated by NKG2D engagement (RSK1/2, p70 S6 kinase, p53, CHK2) was also inhibited by AA (Fig. S8A). These observations support the conclusion that AA exerts profound effects on NKG2D-regulated signal transduction. AA upregulated the phosphorylation of two kinases not responsive to NKG2D stimulation (Fig. S8B), while 12 phosphorylation sites across 11 proteins were unaffected by either NKG2D engagement or AA (Fig. S8C).

To validate the impact of AA on NKG2D-dependent ERK phosphorylation in a different experimental system, we used NK92 cells stimulated with the NKG2D ligand MICA [71]. Figure 6C and D show that a 20-minute exposure of NK92 cells to immobilized MICA prompted an approximately 2.5-fold increase in ERK phosphorylation. Preincubation with AA completely abrogated this pERK induction, which can presumably be attributed, at least partially, to AA’s suppressive action on NKG2D surface expression.

**Fig. 7:**
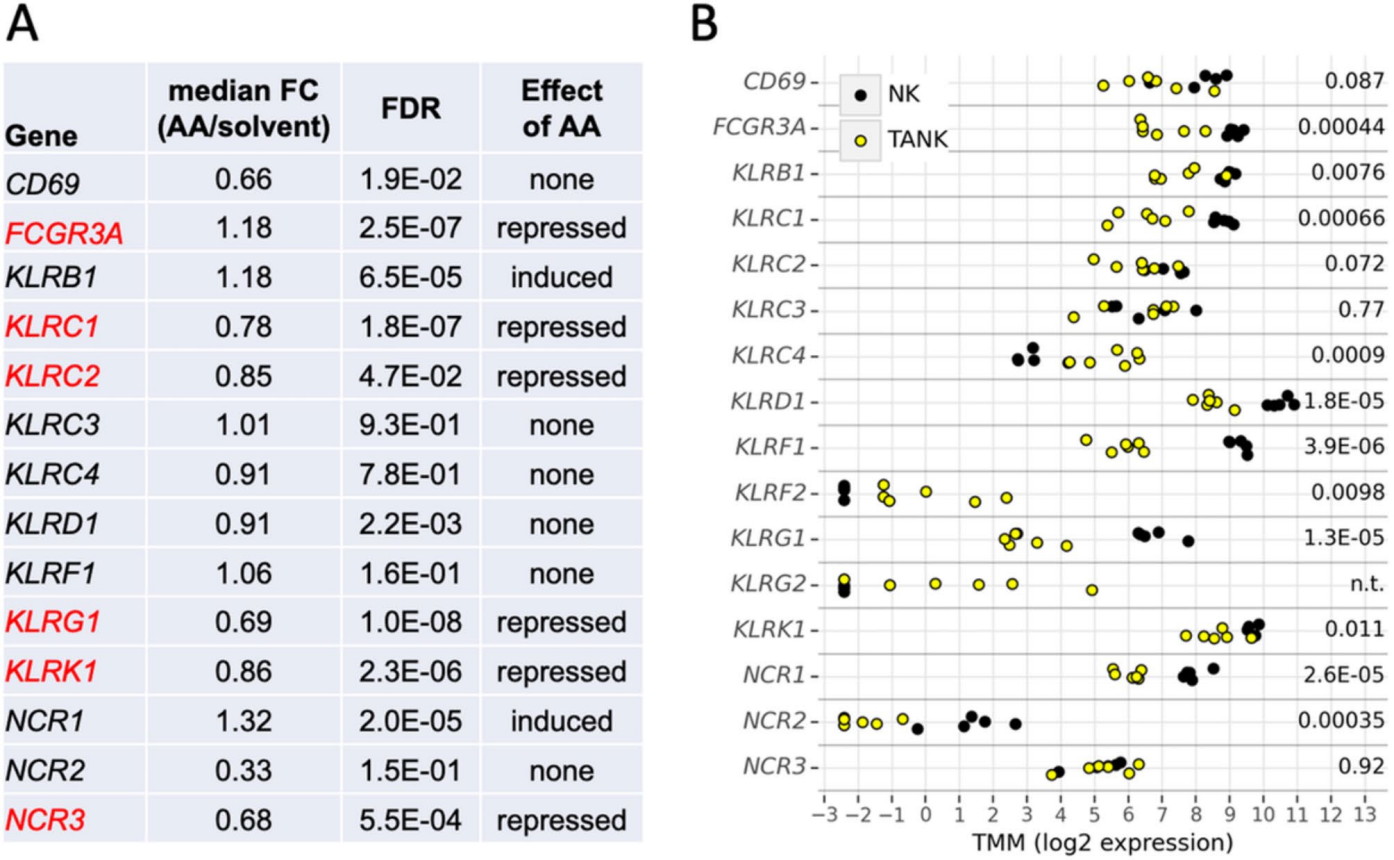
Regulation of activating NK cell receptor genes by AA and the OC TME. **(A)** RNA-Seq data of primary NK cells treated with 50 µM AA or solvent for 3.5 h as in Fig. 3. The table shows the median FC and FDR for *n* = 4 biological replicates, organized in alphabetical order by gene names. **(B)** Expression of the same genes as in panel A in primary NK cells and tumor-associated NK cells (TANK) isolated form the ascites of OC patients. RNA-Seq was performed with *n* = 4 NK cell samples (black dots) and *n* = 6 TANK samples (yellow circles). CPM values were transformed by scaling normalization (Trimmed Mean of M-values, TMM) [49]. The numbers on the right denote the FDR values (NK versus TANK)

Further analysis of the RNA-Seq data (Fig. 7A; Table S3) identified a subset of six activating NK cell receptor genes, i.e., *FCGR3A* (CD16), *KLRC1/C2/G1*, *KLRK1* (NKG2D) and *NCR3* (NKp30), that are repressed by AA treatment. This suggests that besides mechanisms directly affecting protein expression and/or function, transcriptional repression may also be involved in the AA-triggered inhibition of activating NK cell receptor surface expression. Consistent with this hypothesis, AA induced *NCR1* (NKp46) gene expression, but reduced NKp46 surface levels (Fig. 6A).

### Potential clinical associations of activating NK cell receptors

To assess the potential clinical relevance of these findings we investigated whether the AA-induced changes in the expression of activating NK cell receptor genes coincide with the altered transcriptional profile of NK cells in OC ascites, referred to as tumor-associated NK cells (TANK). As shown by the comparative RNA-Seq analysis in Fig. 7B and Table S5, primary NK cells from healthy donors exhibited significantly higher levels of multiple activating NK cell receptor genes compared to TANK, including FCGR3A, KLRK1, KLRC1 and KLRCG1. For *NCR1-3* we did not observe such alignment, which we attribute to the influence of mediators in the TME other than AA on the transcriptome of TANK. Notably, we did not detect any significant changes in the expression of inhibitory NK cell receptor genes of the KIR family (Table S5). This observation suggests that the impact of the TME, including AA, on activating receptors plays a dominant role in the dysregulation of TANK.

**Fig. 8:**
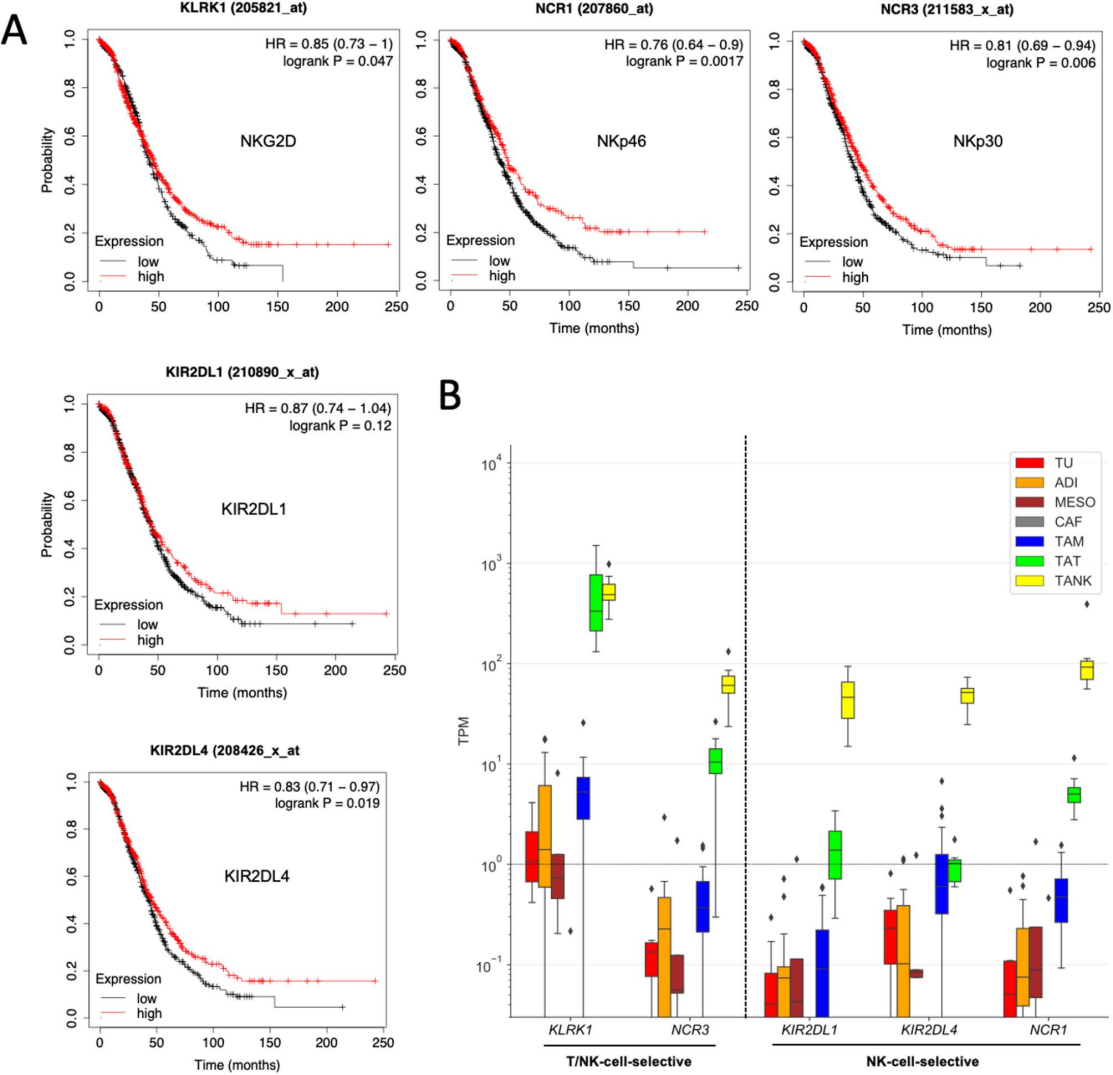
Expression of activating NK cell receptor genes is associated with poor survival of OC. **(A)** Kaplan-Meier plots showing the association of OC overall survival with mRNA expression of the indicated NK cell receptors (plots generated by the KMP database). HR: hazard ratio. **(B)** Expression of the genes analyzed in panel A in different cell types of the OC TME. TU: tumor cells; ADI: adipocytes; MESO: mesothelial cells; CAF: carcinoma-associated fibroblasts; TAM: tumor-associated macrophages; TAT: tumor-associated T cells; TANK: tumor-associated NK cells. TU, ADI, MESO, CAF and TAM were isolated from omental metastases of OC patients, and TAT and TANK from OC ascites. The plot is based on our previously published RNA-Seq data [35]. Box plots show the median (line), upper and lower quartiles (box), range (whiskers) and outliers (diamonds)

Next, we examined the association between expression of activating NK cell receptor genes and the clinical course of OC. The Kaplan-Meier plots depicted in Fig. 8A reveal that low expression levels of four genes — *KLRK1*, *NCR1*, *NCR3* and *KIR2DL4* (CD158D) — is significantly correlated with reduced overall survival in OC patients. Analysis of our previously published RNA-Seq dataset [35], which includes various cell types found in OC ascites and omental metastases, showed selective expression of *KIR2DL1*, *KIR2DL4*, and *NCR1* in tumor-associated NK cells (TANK), and *KLRK1* and *NCR3* in both TANK and tumor-associated T cells (TAT) (Fig. 8B). These observations suggest that low expression of activating NK cell receptors is linked to a poor clinical outcome of OC.

### Inhibition of IL-2-dependent NK cell proliferation and cytotoxicity

**Fig. 9:**
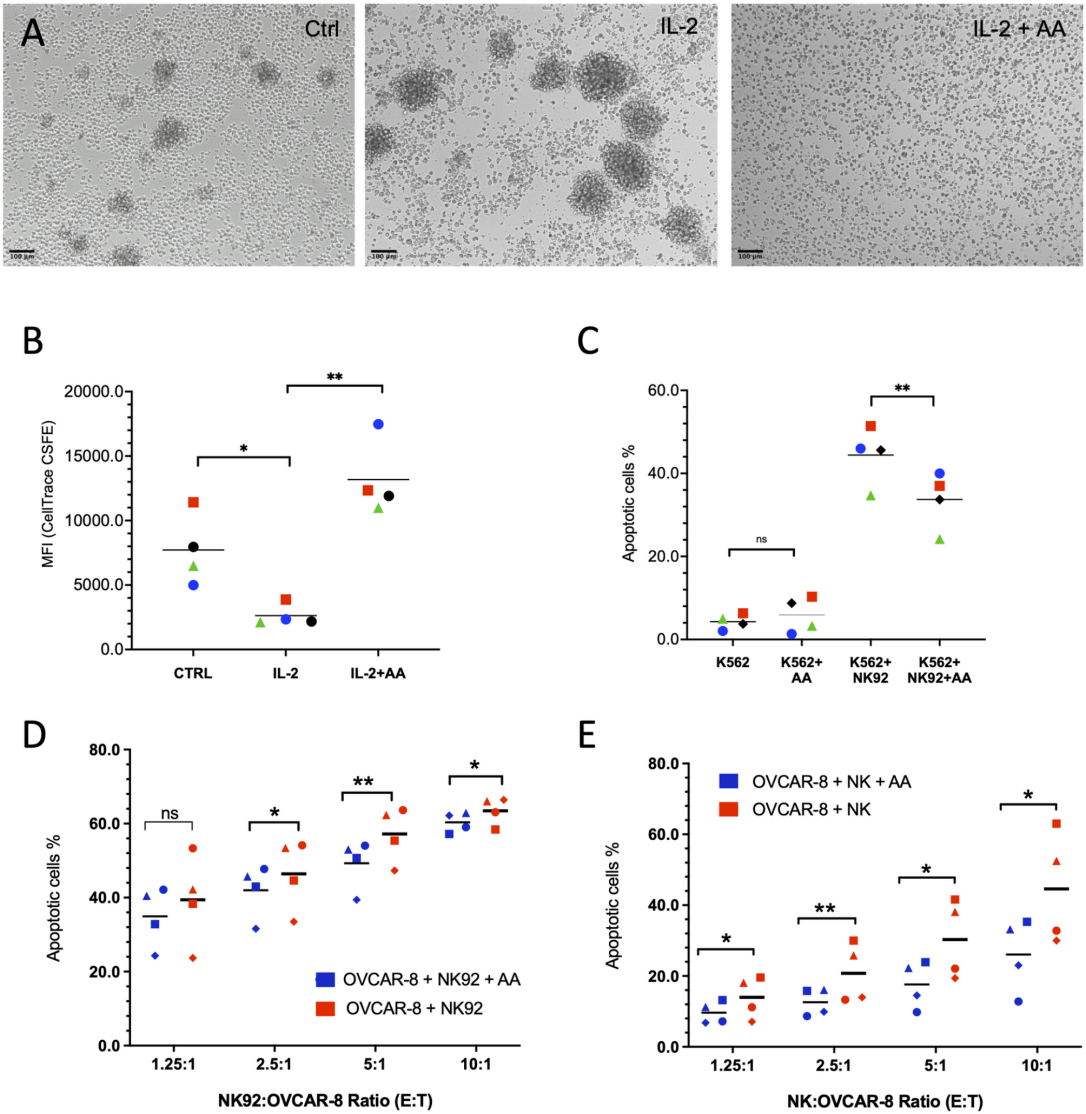
AA inhibits IL-2-dependent NK cell proliferation and cytotoxicity. **(A)** NK92 cells were incubated with AA, IL-2 or IL-2 plus AA. Bright field microscopic pictures were taken after 72 h. **(B)** Quantitative evaluation by flow cytometry of *n* = 4 biological replicates treated as in panel A and stained with CellTrace™ CFSE. Cells were analyzed after 3 days (see Fig. S9 for a representative histogram). **p < 0.01; *p < 0.05 by paired t test. The decreased numbers of AA-treated cells are not due to the induction of cell death (see Fig. S10). **(C)** NK92 were incubated with K562 target cells (ratio 4:1) for 3 h and the percentage of apoptotic cells was determined by PI staining and gating for K562 cells (see Fig. S11 for representative scatter plots). **(D**,**E)** NK92 cells (D) and primary NK cells (E) were incubated with OVCAR-8 as target cells at different effector to target cell (E: T) ratios for 3 h. The percentage of apoptotic OVCAR-8 cells was determined as in panel C. The data in panels B-E are based on *n* = 4 biological replicates each indicated by different symbols. Mean values are indicated by horizontal lines. *p < 0.05; **p < 0.01; ns: not significant by paired t-test

Finally, we assessed the influence of AA on biological functions of NK cells, focusing specifically on cell proliferation and cytotoxicity. NK92 cells, when stimulated with IL-2, exhibited notable cell growth (Fig. 9A), equating to an approximately 3-fold increase in cell numbers over three days, as measured by flow cytometry of cells stained with CellTrace™ CFSE (Fig. 9B and S9). Remarkably, AA nullified IL-2-induced cell growth, reducing it to a level lower than that observed in control cells not treated with IL-2 (Fig. 9A, B). Quantitative analysis revealed a difference of approximately 3 population doublings between cell treated with IL-2 or IL-2 plus AA (Fig. 9B). Propidium iodide staining showed no significant effect of AA (Fig. S10), indicating that the reduced cell numbers in the assay in Fig. 9A and B are not due to AA-induced cell death.

To evaluate their cytotoxic capabilities, NK92 cells were co-cultured with K562 target cells, and the proportion of apoptotic K562 cells was determined by flow cytometry of propidium-iodide-stained cells (Fig. S11). As depicted in Figs. 9C and 44% of K562 cells co-cultured with NK92 cells underwent apoptosis within 3 h, marking an 11-fold increase as compared to control K562 cells alone. This percentage significantly dropped to 34% in the presence of AA. Consistent with this finding, AA also reduced the killing of the OC cell line OVCAR-8 by NK92 (Fig. 9D) as well as primary NK cells (Fig. 9E), which was statistically significant at effector to target cell ratios ≥ 1.25 (NK92) and ≥ 2.5 (primary NK cells), respectively.

Taken together, these findings underscore that AA’s modulation of signal transduction pathways in NK cells yields a robust decrease of proliferation and also inhibits cytotoxicity, thereby diminishing their anti-tumor activity.

## Discussion

Ovarian cancer employs several mechanisms to evade NK cell-mediated cytotoxicity. These include the downregulation of NKG2D ligands [14, 17] and the increased expression of inhibitory ligands such as HLA-E [72, 73]. Additionally, exhaustion receptors TIM-3 and PD-1 may be upregulated on lymphocytes [74]. The secretion of immunosuppressive factors like TGF-β also counteracts the activity of NK cell cytotoxicity receptors [16, 75]. However, the impact of AA on the balance of activating and inhibitory receptor expression, signaling and function remains poorly understood. This issue has been investigated in the present study and will be discussed in detail in the following sections.

### Production of cytotoxic ROS by NK cells

ROS play a critical role in the immune response, but they also have the potential to damage proteins, lipids, DNA, and disrupt signaling pathways [76, 77]. Consequently, maintaining precise control over ROS levels is essential. This regulation is achieved through the cell’s inherent detoxification systems. Central to this process are superoxide dismutases, catalase, glutathione peroxidases and peroxiredoxins, which collectively neutralize ROS and safeguard cellular integrity [76, 77]. This antioxidant system is governed by a negative feedback loop, wherein increased ROS levels trigger the expression of genes coding for ROS-detoxifying proteins, with the transcription factor NRF2 playing a pivotal role [78].

A prominent aspect of the RNA-Seq data presented above is an upregulation of genes involved in ROS detoxification, as revealed by DAVID-based functional annotation. This includes several downstream targets the NRF2 transcription factor (*NFE2L2* gene), including glutathione reductase, peroxiredoxins, superoxide dismutase, thioredoxins as well as thioredoxin reductase. The NRF2 complex, which is activated in response to ROS, serves as a master regulator of genes encoding components of the antioxidant defense system. The observed upregulation of ROS detoxification genes in our study likely stems from a rapid increase in ROS production upon exposure of NK cells to AA.

The generation of ROS by AA has been described previously and involves a complex interplay of metabolic pathways and the activation of specific enzymes and cellular processes [76]. This multifaceted mechanism includes (i) AA metabolism via cyclooxygenases, lipoxygenases and cytochrome P450 enzymes, where electron transfer can result in the generation of ROS as byproducts [79, 80]; (ii) the stimulation of the plasma-membrane-bound NADPH oxidase complex by AA [81, 82]; and (iii) the disturbance of mitochondrial electron transport chain functionality by causing electron leakage [83]. The extent to which these mechanisms contribute to ROS generation in AA-treated NK cells remains unclear. However, our findings indicate that, likely through an initial surge in ROS levels, AA triggers the activation of the ROS detoxification system. This, in turn, potentially compromises the ROS-dependent cytotoxic function of NK cells.

### Signaling via activating cytokine and NK cell receptors

IL-2 and IL-15 are crucial cytokines for the development, proliferation and function of NK cells [7, 84]. Despite their distinct roles in the immune system, they share several signaling components, primarily due to use of common receptor subunits and signaling molecules. IL-2 and IL-15 receptors share the β and γ subunits [85]. The engagement of IL-2 or IL-15 with their receptors initiates the recruitment of JAK1 and JAK3 to these common subunits, resulting in the phosphorylation of STAT1 and STAT5, which triggers their translocation to the nucleus and the activation of specific target genes [86]. Our phosphoproteome and transcriptome analyses highlight the suppression of cytokine signaling as a key effect of AA on NK cells. Notably, our findings demonstrate that AA represses the expression of IL-2 target genes and disrupts signal transduction pathways initiated by IL-2 and IL-15 receptors through inhibition of STAT1 phosphorylation. The relevance of this finding is underscored by a previous phosphoproteomic analysis of NK92 cells stimulated with IL-2 or IL-15, which revealed STAT1-mediated activation to p90 ribosomal S6 kinase (p90RSK) as a functionally crucial signaling event triggered by both ligands [87].

Previous studies have shown that elevated concentrations of PUFAs, such as AA, disrupt the structure of lipid rafts in the plasma membrane [88–92]. This disruption can impair the functionality of receptors and associated proteins that critically rely on localization within lipid rafts for proper function. Cytokine-signaling-related proteins associated with lipid rafts [92] include, for example, IL-2 and IL-15 receptor subunits in T cells [93–95], interferon receptors and STAT1 in macrophages [39], STAT1 and STAT3 in hepatoma cells [96]. This suggests that the inhibitory impact of AA on IL-2 and IL-15 signaling may, at least in part, be mediated through disruption of compartmentalization of the respective receptor subunits and STAT proteins within lipid rafts.

In addition to cytokine receptors, AA also disrupted the functionality of activating NK cell receptors. This is evident from the decreased IL-2-induced surface expression of these receptors and the diminished phosphorylation of signaling proteins, such as pERK and pSTAT1, following the engagement of NKG2D by an activating antibody or MICA. Activation of NKG2D triggers a series of signaling pathways distinct from that triggered by cytokine receptors [97]. However, a shared characteristic with IL-2 and IL-15 receptors is the essential compartmentalization of NKG2D within lipid rafts for its functional activity [98–100]. Therefore, it is conceivable that the disruption of lipid rafts represents a common mechanism undermining the functionality of both cytokine and NK cell receptors. As indicated by or RNA-Seq dataset, several activating NK cell receptor genes, including *KLRK1* and *NCR3*, are down-regulated by AA. This observation implies that the primary mechanism affecting activating NK cell receptors involves alterations in protein expression and/or spatial organization within the plasma membrane, which in some cases may be enhanced by transcriptional repression.

We have previously reported that AA interferes with the function of macrophages in the TME by two fundamentally different mechanisms: (i) induction of Ca^2+^-dependent p38 pathway linked to deregulation of RHO-GTPases, impaired actin filament organization and augmented release of exosomes [38], and (ii) impairment of pro-inflammatory signal transduction due to impaired JAK/STAT signaling caused by AA-mediated perturbation of lipid rafts, which are crucial for the spatial organization of cytokine receptors and associated signaling proteins in the plasma membrane [39]. Notably, the first mechanism is triggered by AA at concentrations as low as 5 µM [38], whereas the disruption of lipid rafts occurs at significantly higher AA concentrations, around 50 µM [39]. Importantly, AA levels exceeding the concentration used in the present study (50 µM) are observed in a subset of OC patients and correlate with poorer clinical outcomes [40].

### Functional and potential clinical relevance

Since AA impairs the signaling by activating cytokine and NK cell receptors crucial to NK cell proliferation and cytotoxicity, we explored this connection through functional assays. These studies revealed that AA blocks both basal and IL-2-induced proliferation of NK cells and inhibits cytotoxicity towards targets cells (K562, OVCAR-8). K562 cells are an established standardized target for evaluating the functional capacity of NK cells due to their high expression of activating NK cell receptor ligands, the absence of MHC class I molecules and a high reproducibility of the assay [101]. Importantly, AA also reduced killing activity towards OVCAR-8, suggesting that the AA-mediated restriction of NK cell activities is relevant for NK cell-dependent immunosurveillance in OC.

Our data suggest that the impact of AA on central signal transduction pathways has significant implications for the biological functions of NK cells. This hypothesis is supported by several observations: First, according to RNA-Seq analysis several activating NK cell receptor genes that are repressed by AA also exhibit a significantly lower expression in TANK from OC ascites compared to normal NK cells, including *FCGR3* (CD16) and *KLRK1* (NKG2D). Second, the expression of multiple genes encoding NK cell receptors, including *NKG2D* and *NCR3* (NKp30), is associated with a short overall survival of OC. This is consistent with previous studies that have identified a similar clinical correlation for soluble NKG2D ligands [14, 15] and B7-H6, a ligand for NKp30 [102]. Third, AA additionally inhibits the expression of genes coding for chemokine and cytokine receptors essential for anti-tumor NK cell functions, notably CX3CR1 and CXCR3, which are similarly downregulated in TANK compared to normal NK cells (Table S5).

Clarifying which precise impacts of AA on the NK cell signaling network are pivotal for the clinical progression of OC remains a pivotal challenge. However, AA’s impact on cytokine and NK cell receptor functions is likely to be among the primary contributors. In this context, it will be of paramount importance to elucidate the precise mechanisms underlying this defect, serving as a foundation to prevent or mitigate the adverse effects of AA.

## Conclusion

**Fig. 10:**
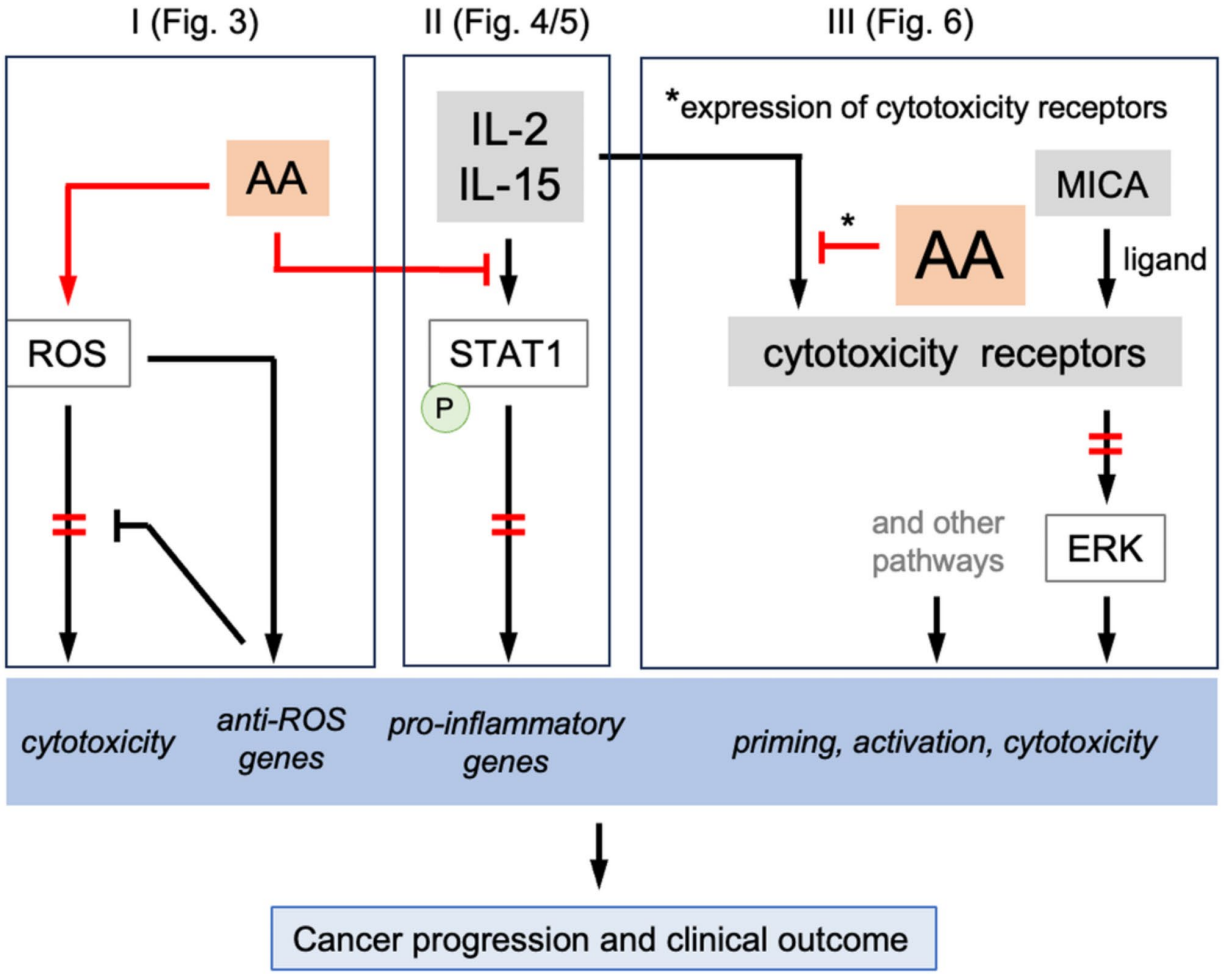
Model summarizing the effects of AA on signal transduction pathways and their functional consequences. Our data suggest different mechanisms AA utilizes to interfere with signal transduction in NK cells, thereby impairing their role in anti-tumor surveillance and resulting a poor clinical outcome. This model encompasses multiple potential mechanisms. **I**: AA triggers a burst of ROS, which in turn upregulates ROS-detoxifying genes, resulting in decreased cytotoxic ROS levels (data in Fig. 3). **II**: AA inhibits signal transduction by NK-cell-activating cytokines (such as IL-2 and IL-15) through interference with STAT1 phosphorylation (Figs. 4 and 5). **III**: AA inhibits the cytokine-induced expression of surface expression of several cytotoxicity receptors, including NKG2D, and interferes with ERK phosphorylation induced by the NKG2D-ligand MICA (Fig. 6). AA presumably also affects other receptors (Fig. 6A) and pro-inflammatory signaling pathways (Figs. 1, 2 and 3) involved in NK cell activation and/or cytotoxicity

Our findings reveal that AA disrupts signal transduction through several mechanisms, impacting diverse pathways as summarized in Fig. 10. These mechanisms include: (i) the upregulation of ROS-detoxifying genes, which may lead to a reduction in cytotoxic ROS levels; (ii) the suppression of signaling by activating cytokines, accompanied by a decrease in STAT1 phosphorylation; and (iii) the disruption of surface receptor expression including NCRs and NKG2D corresponding with reduced phosphorylation of downstream signaling molecules. Functionally, these alterations likely cooperate to impair proliferation, cytotoxicity and cytokine expression (including TNFα and IFNγ). As the expression of NKG2D and other NK cell receptors is inversely associated with the survival of OC, these observations point to a functional link between AA, NK cell dysfunction and OC progression.

## Electronic supplementary material

Below is the link to the electronic supplementary material.

Supplementary Material 1

Supplementary Material 2

## Data Availability

RNA-Seq data were deposited at EBI ArrayExpress (accession numbers E-MTAB-13971, E-MTAB-14053). Mass spectrometric raw data and settings were uploaded with the to the ProteomeXchange Consortium with dataset (identifier: PXD049958).

## Abbreviations

AA: Arachidonic Acid
ADI: Adipocyte
CAF: Carcinoma-Associated Fibroblast
CPDB: ConsensusPathDB
CPM: Counts Per Million
DDA: Data-Dependent Mode
FC: Fold Change
FDR: False Discovery Rate
HPLC: High-Performance Liquid Chromatography
MESO: Mesothelial Cell
MS: Mass Spectrometry
MHC: Major Histocompatibility Complex
NAC: N-Acetylcysteine
NK cell: Natural Killer Cell
OC: Ovarian Cancer
PUFA: Polyunsaturated Fatty Acid
RNA-Seq: RNA Sequencing
ROS: Reactive Oxygen Species
RT-qPCR: Quantitative Reverse Transcriptase Polymerase Chain Rection
RSLC: Rapid Separation Liquid Chromatography
TAM: Tumor-Associated Macrophages
TAT: Tumor-Associated T cells
TANK: Tumor-Associated NK cell
TMT: Tandem Mass Tag
TEAB: Triethylammonium Bicarbonate
TME: Tumor Microenvironment
TU: Tumor Cell

## References

[1] Stokic-Trtica V, Diefenbach A, Klose CSN. NK Cell Development in Times of Innate Lymphoid Cell Diversity. Front Immunol. 2020;11:813. 32733432 10.3389/fimmu.2020.00813 PMC7360798

[2] Chan IS, Ewald AJ. The changing role of natural killer cells in cancer metastasis. J Clin Invest. 2022;132. 10.1172/JCI143762 PMC8920322 35289318

[3] Portale F, Di Mitri D. NK cells in Cancer: mechanisms of dysfunction and therapeutic potential. Int J Mol Sci. 2023;24:9521. 37298470 10.3390/ijms24119521 PMC10253405

[4] Wolf NK, Kissiov DU, Raulet DH. Roles of natural killer cells in immunity to cancer, and applications to immunotherapy. Nat Rev Immunol. 2023;23:90–105. 35637393 10.1038/s41577-022-00732-1

[5] Sivori S, Vacca P, Del Zotto G, Munari E, Mingari MC, Moretta L. Human NK cells: surface receptors, inhibitory checkpoints, and translational applications. Cell Mol Immunol. 2019;16:430–41. 30778167 10.1038/s41423-019-0206-4 PMC6474200

[6] Ramirez-Labrada A, Pesini C, Santiago L, Hidalgo S, Calvo-Perez A, Onate C, et al. All about (NK Cell-Mediated) death in two acts and an unexpected encore: initiation, execution and activation of adaptive immunity. Front Immunol. 2022;13:896228. 35651603 10.3389/fimmu.2022.896228 PMC9149431

[7] Zwirner NW, Domaica CI. Cytokine regulation of natural killer cell effector functions. BioFactors. 2010;36:274–88. 20623510 10.1002/biof.107

[8] Paul S, Lal G. The molecular mechanism of natural killer cells function and its importance in Cancer Immunotherapy. Front Immunol. 2017;8:1124. 28955340 10.3389/fimmu.2017.01124 PMC5601256

[9] Corvino D, Kumar A, Bald T. Plasticity of NK cells in Cancer. Front Immunol. 2022;13:888313. 35619715 10.3389/fimmu.2022.888313 PMC9127295

[10] Konjevic GM, Vuletic AM, Mirjacic Martinovic KM, Larsen AK, Jurisic VB. The role of cytokines in the regulation of NK cells in the tumor environment. Cytokine. 2019;117:30–40. 30784898 10.1016/j.cyto.2019.02.001

[11] Groh V, Wu J, Yee C, Spies T. Tumour-derived soluble MIC ligands impair expression of NKG2D and T-cell activation. Nature. 2002;419:734–8. 12384702 10.1038/nature01112

[12] Xing S, de Ferrari L. NKG2D and MICA/B shedding: a ‘tag game’ between NK cells and malignant cells. Clin Transl Immunol. 2020;9:e1230. 10.1002/cti2.1230 PMC7754731 33363734

[13] Zingoni A, Vulpis E, Loconte L, Santoni A. NKG2D ligand shedding in response to stress: role of ADAM10. Front Immunol. 2020;11:447. 32269567 10.3389/fimmu.2020.00447 PMC7109295

[14] Vyas M, Reinartz S, Hoffmann N, Reiners KS, Lieber S, Jansen JM, et al. Soluble NKG2D ligands in the ovarian cancer microenvironment are associated with an adverse clinical outcome and decreased memory effector T cells independent of NKG2D downregulation. Oncoimmunology. 2017;6:e1339854. 28932639 10.1080/2162402X.2017.1339854 PMC5599084

[15] Tan G, Spillane KM, Maher J. The role and regulation of the NKG2D/NKG2D ligand system in Cancer. Biology (Basel). 2023;12. 10.3390/biology12081079 PMC10452210 37626965

[16] Lazarova M, Steinle A. Impairment of NKG2D-Mediated tumor immunity by TGF-beta. Front Immunol. 2019;10:2689. 31803194 10.3389/fimmu.2019.02689 PMC6873348

[17] Krockenberger M, Dombrowski Y, Weidler C, Ossadnik M, Honig A, Hausler S, et al. Macrophage migration inhibitory factor contributes to the immune escape of ovarian cancer by down-regulating NKG2D. J Immunol. 2008;180:7338–48. 18490733 10.4049/jimmunol.180.11.7338 PMC3607742

[18] Krockenberger M, Kranke P, Hausler S, Engel JB, Horn E, Nurnberger K, et al. Macrophage migration-inhibitory factor levels in serum of patients with ovarian cancer correlates with poor prognosis. Anticancer Res. 2012;32:5233–8. 23225421

[19] Li X, Liu Y, Zheng S, Zhang T, Wu J, Sun Y, et al. Role of exosomes in the immune microenvironment of ovarian cancer. Oncol Lett. 2021;21:377. 33777201 10.3892/ol.2021.12638 PMC7988709

[20] Chung DC, Ghaedi M, Warner K, Sayad A, Saibil SD, Bernardini MQ, et al. Characterization of innate lymphoid cell subsets infiltrating melanoma and epithelial ovarian tumors. Oncoimmunology. 2024;13:2349347. 38746870 10.1080/2162402X.2024.2349347 PMC11093043

[21] Wong JL, Berk E, Edwards RP, Kalinski P. IL-18-primed helper NK cells collaborate with dendritic cells to promote recruitment of effector CD8 + T cells to the tumor microenvironment. Cancer Res. 2013;73:4653–62. 23761327 10.1158/0008-5472.CAN-12-4366 PMC3780558

[22] Kumar S. Natural killer cell cytotoxicity and its regulation by inhibitory receptors. Immunology. 2018;154:383–93. 29512837 10.1111/imm.12921 PMC6002213

[23] Raulet DH. Missing self recognition and self tolerance of natural killer (NK) cells. Semin Immunol. 2006;18:145–50. 16740393 10.1016/j.smim.2006.03.003

[24] Cruz-Munoz ME, Valenzuela-Vazquez L, Sanchez-Herrera J, Santa-Olalla Tapia J. From the missing self hypothesis to adaptive NK cells: insights of NK cell-mediated effector functions in immune surveillance. J Leukoc Biol. 2019;105:955–71. 30848847 10.1002/JLB.MR0618-224RR

[25] Shifrin N, Raulet DH, Ardolino M. NK cell self tolerance, responsiveness and missing self recognition. Semin Immunol. 2014;26:138–44. 24629893 10.1016/j.smim.2014.02.007 PMC3984600

[26] Pegram HJ, Andrews DM, Smyth MJ, Darcy PK, Kershaw MH. Activating and inhibitory receptors of natural killer cells. Immunol Cell Biol. 2011;89:216–24. 20567250 10.1038/icb.2010.78

[27] Gaggero S, Witt K, Carlsten M, Mitra S. Cytokines orchestrating the natural killer-myeloid cell crosstalk in the Tumor Microenvironment: implications for natural killer cell-based Cancer Immunotherapy. Front Immunol. 2020;11:621225. 33584718 10.3389/fimmu.2020.621225 PMC7878550

[28] Worzfeld T, Finkernagel F, Reinartz S, Konzer A, Adhikary T, Nist A, et al. Proteotranscriptomics Reveal Signaling Networks in the Ovarian Cancer Microenvironment. Mol Cell Proteom. 2018;17:270–89. 10.1074/mcp.RA117.000400 PMC5795391 29141914

[29] Colombo N, Peiretti M, Parma G, Lapresa M, Mancari R, Carinelli S, et al. Newly diagnosed and relapsed epithelial ovarian carcinoma: ESMO Clinical Practice guidelines for diagnosis, treatment and follow-up. Ann Oncol. 2010;21(Suppl 5):v23–30. 20555088 10.1093/annonc/mdq244

[30] Ahmed N, Stenvers KL. Getting to Know Ovarian Cancer ascites: opportunities for targeted therapy-based Translational Research. Front Oncol. 2013;3:256. 24093089 10.3389/fonc.2013.00256 PMC3782691

[31] Worzfeld T, von Pogge E, Huber M, Adhikary T, Wagner U, Reinartz S, et al. The Unique Molecular and Cellular Microenvironment of Ovarian Cancer. Front Oncol. 2017;7:24. 28275576 10.3389/fonc.2017.00024 PMC5319992

[32] Giuntoli RL 2nd, Webb TJ, Zoso A, Rogers O, Diaz-Montes TP, Bristow RE, et al. Ovarian cancer-associated ascites demonstrates altered immune environment: implications for antitumor immunity. Anticancer Res. 2009;29:2875–84. 19661290

[33] Kipps E, Tan DS, Kaye SB. Meeting the challenge of ascites in ovarian cancer: new avenues for therapy and research. Nat Rev Cancer. 2013;13:273–82. 23426401 10.1038/nrc3432 PMC4673904

[34] Almeida-Nunes DL, Mendes-Frias A, Silvestre R, Dinis-Oliveira RJ, Ricardo S. Immune Tumor Microenvironment in Ovarian Cancer ascites. Int J Mol Sci. 2022;23. 10.3390/ijms231810692 PMC9504085 36142615

[35] Sommerfeld L, Finkernagel F, Jansen JM, Wagner U, Nist A, Stiewe T, et al. The multicellular signalling network of ovarian cancer metastases. Clin Transl Med. 2021;11:e633. 34841720 10.1002/ctm2.633 PMC8574964

[36] Kobayashi K, Omori K, Murata T. Role of prostaglandins in tumor microenvironment. Cancer Metastasis Rev. 2018;37:347–54. 29926309 10.1007/s10555-018-9740-2

[37] Kalinski P. Regulation of immune responses by prostaglandin E2. J Immunol. 2012;188:21–8. 22187483 10.4049/jimmunol.1101029 PMC3249979

[38] Dietze R, Hammoud MK, Gomez-Serrano M, Unger A, Bieringer T, Finkernagel F, et al. Phosphoproteomics identify arachidonic-acid-regulated signal transduction pathways modulating macrophage functions with implications for ovarian cancer. Theranostics. 2021;11:1377–95. 33391540 10.7150/thno.52442 PMC7738879

[39] Hammoud MK, Dietze R, Pesek J, Finkernagel F, Unger A, Bieringer T, et al. Arachidonic acid, a clinically adverse mediator in the ovarian cancer microenvironment, impairs JAK-STAT signaling in macrophages by perturbing lipid raft structures. Mol Oncol. 2022;16:3146–66. 35451191 10.1002/1878-0261.13221 PMC9441005

[40] Reinartz S, Finkernagel F, Adhikary T, Rohnalter V, Schumann T, Schober Y, et al. A transcriptome-based global map of signaling pathways in the ovarian cancer microenvironment associated with clinical outcome. Genome Biol. 2016;17:108. 27215396 10.1186/s13059-016-0956-6 PMC4877997

[41] Li K, Mandai M, Hamanishi J, Matsumura N, Suzuki A, Yagi H, et al. Clinical significance of the NKG2D ligands, MICA/B and ULBP2 in ovarian cancer: high expression of ULBP2 is an indicator of poor prognosis. Cancer Immunol Immunother. 2009;58:641–52. 18791713 10.1007/s00262-008-0585-3 PMC11030581

[42] McGilvray RW, Eagle RA, Rolland P, Jafferji I, Trowsdale J, Durrant LG. ULBP2 and RAET1E NKG2D ligands are independent predictors of poor prognosis in ovarian cancer patients. Int J Cancer. 2010;127:1412–20. 20054857 10.1002/ijc.25156

[43] Hughes CS, Moggridge S, Muller T, Sorensen PH, Morin GB, Krijgsveld J. Single-pot, solid-phase-enhanced sample preparation for proteomics experiments. Nat Protoc. 2019;14:68–85. 30464214 10.1038/s41596-018-0082-x

[44] Kamburov A, Wierling C, Lehrach H, Herwig R. ConsensusPathDB–a database for integrating human functional interaction networks. Nucleic Acids Res. 2009;37:D623–8. 18940869 10.1093/nar/gkn698 PMC2686562

[45] Herwig R, Hardt C, Lienhard M, Kamburov A. Analyzing and interpreting genome data at the network level with ConsensusPathDB. Nat Protoc. 2016;11:1889–907. 27606777 10.1038/nprot.2016.117

[46] Robinson MD, McCarthy DJ, Smyth GK. edgeR: a Bioconductor package for differential expression analysis of digital gene expression data. Bioinformatics. 2010;26:139–40. 19910308 10.1093/bioinformatics/btp616 PMC2796818

[47] Love MI, Huber W, Anders S. Moderated estimation of Fold change and dispersion for RNA-seq data with DESeq2. Genome Biol. 2014;15:550. 25516281 10.1186/s13059-014-0550-8 PMC4302049

[48] Sherman BT, Hao M, Qiu J, Jiao X, Baseler MW, Lane HC, et al. DAVID: a web server for functional enrichment analysis and functional annotation of gene lists (2021 update). Nucleic Acids Res. 2022;50:W216–21. 35325185 10.1093/nar/gkac194 PMC9252805

[49] Robinson MD, Oshlack A. A scaling normalization method for differential expression analysis of RNA-seq data. Genome Biol. 2010;11:R25. 20196867 10.1186/gb-2010-11-3-r25 PMC2864565

[50] Safran M, Dalah I, Alexander J, Rosen N, Iny Stein T, Shmoish M, et al. GeneCards Version 3: the human gene integrator. Database (Oxford). 2010;2010:baq020. 20689021 10.1093/database/baq020 PMC2938269

[51] Stelzer G, Rosen N, Plaschkes I, Zimmerman S, Twik M, Fishilevich S, et al. Curr Protoc Bioinf. 2016;54(1 30 1–1):3033. The GeneCards Suite: From Gene Data Mining to Disease Genome Sequence Analyses. 10.1002/cpbi.5 27322403

[52] Ben-Shmuel A, Sabag B, Biber G, Barda-Saad M. The role of the Cytoskeleton in regulating the natural killer cell Immune Response in Health and Disease: from Signaling dynamics to function. Front Cell Dev Biol. 2021;9:609532. 33598461 10.3389/fcell.2021.609532 PMC7882700

[53] Zhu Y, Xie J, Shi J. Rac1/ROCK-driven membrane dynamics promote natural killer cell cytotoxicity via granzyme-induced necroptosis. BMC Biol. 2021;19:140. 34325694 10.1186/s12915-021-01068-3 PMC8323222

[54] Parameswaran R, Ramakrishnan P, Moreton SA, Xia Z, Hou Y, Lee DA, et al. Repression of GSK3 restores NK cell cytotoxicity in AML patients. Nat Commun. 2016;7:11154. 27040177 10.1038/ncomms11154 PMC4822012

[55] Cichocki F, Valamehr B, Bjordahl R, Zhang B, Rezner B, Rogers P, et al. GSK3 inhibition drives maturation of NK cells and enhances their Antitumor activity. Cancer Res. 2017;77:5664–75. 28790065 10.1158/0008-5472.CAN-17-0799 PMC5645243

[56] Bariagaber AK, Whalen MM. Decreased adenylyl cyclase and cAMP-dependent protein kinase activities inhibit the cytotoxic function of human natural killer cells. Hum Immunol. 2003;64:866–73. 12941541 10.1016/s0198-8859(03)00154-x

[57] Whalen MM, Odman-Ghazi SO. Effects of adenylyl cyclase and protein kinase a inhibition on signaling enzymes in natural killer cells: comparison to tributyltin. Hum Exp Toxicol. 2006;25:333–40. 16866191 10.1191/0960327106ht630oa

[58] Brillantes M, Beaulieu AM. Transcriptional control of natural killer cell differentiation. Immunology. 2019;156:111–9. 30450565 10.1111/imm.13017 PMC6329199

[59] Goh W, Sudholz H, Foroutan M, Scheer S, Pfefferle A, Delconte RB, et al. IKAROS and AIOLOS directly regulate AP-1 transcriptional complexes and are essential for NK cell development. Nat Immunol. 2024;25:240–55. 38182668 10.1038/s41590-023-01718-4

[60] Aramburu J, Azzoni L, Rao A, Perussia B. Activation and expression of the nuclear factors of activated T cells, NFATp and NFATc, in human natural killer cells: regulation upon CD16 ligand binding. J Exp Med. 1995;182:801–10. 7650486 10.1084/jem.182.3.801 PMC2192167

[61] Brand A, Singer K, Koehl GE, Kolitzus M, Schoenhammer G, Thiel A, et al. LDHA-Associated Lactic Acid Production blunts Tumor Immunosurveillance by T and NK Cells. Cell Metab. 2016;24:657–71. 27641098 10.1016/j.cmet.2016.08.011

[62] Lai CB, Mager DL. Role of runt-related transcription factor 3 (RUNX3) in transcription regulation of natural cytotoxicity receptor 1 (NCR1/NKp46), an activating natural killer (NK) cell receptor. J Biol Chem. 2012;287:7324–34. 22253448 10.1074/jbc.M111.306936 PMC3293567

[63] Lotem J, Levanon D, Negreanu V, Leshkowitz D, Friedlander G, Groner Y. Runx3-mediated transcriptional program in cytotoxic lymphocytes. PLoS ONE. 2013;8:e80467. 24236182 10.1371/journal.pone.0080467 PMC3827420

[64] Levanon D, Negreanu V, Lotem J, Bone KR, Brenner O, Leshkowitz D, et al. Transcription factor Runx3 regulates interleukin-15-dependent natural killer cell activation. Mol Cell Biol. 2014;34:1158–69. 24421391 10.1128/MCB.01202-13 PMC3958033

[65] Mi H, Ebert D, Muruganujan A, Mills C, Albou LP, Mushayamaha T, et al. PANTHER version 16: a revised family classification, tree-based classification tool, enhancer regions and extensive API. Nucleic Acids Res. 2021;49:D394–403. 33290554 10.1093/nar/gkaa1106 PMC7778891

[66] Mi H, Muruganujan A, Casagrande JT, Thomas PD. Large-scale gene function analysis with the PANTHER classification system. Nat Protoc. 2013;8:1551–66. 23868073 10.1038/nprot.2013.092 PMC6519453

[67] Kryszczuk M, Kowalczuk O. Significance of NRF2 in physiological and pathological conditions an comprehensive review. Arch Biochem Biophys. 2022;730:109417. 36202215 10.1016/j.abb.2022.109417

[68] Ran GH, Lin YQ, Tian L, Zhang T, Yan DM, Yu JH, et al. Natural killer cell homing and trafficking in tissues and tumors: from biology to application. Signal Transduct Target Ther. 2022;7:205. 35768424 10.1038/s41392-022-01058-z PMC9243142

[69] Henney CS, Kuribayashi K, Kern DE, Gillis S. Interleukin-2 augments natural killer cell activity. Nature. 1981;291:335–8. 6164929 10.1038/291335a0

[70] Wensveen FM, Jelencic V, Polic B. NKG2D: a Master Regulator of Immune Cell responsiveness. Front Immunol. 2018;9:441. 29568297 10.3389/fimmu.2018.00441 PMC5852076

[71] Lopez-Soto A, Huergo-Zapico L, Acebes-Huerta A, Villa-Alvarez M, Gonzalez S. NKG2D signaling in cancer immunosurveillance. Int J Cancer. 2015;136:1741–50. 24615398 10.1002/ijc.28775

[72] Zheng H, Lu R, Xie S, Wen X, Wang H, Gao X, et al. Human leukocyte antigen-E alleles and expression in patients with serous ovarian cancer. Cancer Sci. 2015;106:522–8. 25711417 10.1111/cas.12641 PMC4452152

[73] Andersson E, Poschke I, Villabona L, Carlson JW, Lundqvist A, Kiessling R, et al. Non-classical HLA-class I expression in serous ovarian carcinoma: correlation with the HLA-genotype, tumor infiltrating immune cells and prognosis. Oncoimmunology. 2016;5:e1052213. 26942060 10.1080/2162402X.2015.1052213 PMC4760332

[74] Song Z, Zhang J, Sun Y, Jiang Z, Liu X. Establishment and validation of an immune infiltration predictive model for ovarian cancer. BMC Med Genomics. 2023;16:227. 37759229 10.1186/s12920-023-01657-x PMC10538244

[75] Trotta R, Dal Col J, Yu J, Ciarlariello D, Thomas B, Zhang X, et al. TGF-beta utilizes SMAD3 to inhibit CD16-mediated IFN-gamma production and antibody-dependent cellular cytotoxicity in human NK cells. J Immunol. 2008;181:3784–92. 18768831 10.4049/jimmunol.181.6.3784 PMC2924753

[76] Auten RL, Davis JM. Oxygen toxicity and reactive oxygen species: the devil is in the details. Pediatr Res. 2009;66:121–7. 19390491 10.1203/PDR.0b013e3181a9eafb

[77] Birben E, Sahiner UM, Sackesen C, Erzurum S, Kalayci O. Oxidative stress and antioxidant defense. World Allergy Organ J. 2012;5:9–19. 23268465 10.1097/WOX.0b013e3182439613 PMC3488923

[78] Averill-Bates D. Reactive oxygen species and cell signaling. Rev Biochim Biophys Acta Mol Cell Res. 2024;1871:119573. 10.1016/j.bbamcr.2023.119573 37949302

[79] Zangar RC, Davydov DR, Verma S. Mechanisms that regulate production of reactive oxygen species by cytochrome P450. Toxicol Appl Pharmacol. 2004;199:316–31. 15364547 10.1016/j.taap.2004.01.018

[80] Kwon KJ, Jung YS, Lee SH, Moon CH, Baik EJ. Arachidonic acid induces neuronal death through lipoxygenase and cytochrome P450 rather than cyclooxygenase. J Neurosci Res. 2005;81:73–84. 15931672 10.1002/jnr.20520

[81] Hartfield PJ, Robinson JM. Arachidonic acid activates NADPH oxidase by a direct, calmodulin-regulated mechanism. Prostaglandins Other Lipid Mediat. 1998;56:1–6. 9674016 10.1016/s0090-6980(98)00036-7

[82] Shiose A, Sumimoto H. Arachidonic acid and phosphorylation synergistically induce a conformational change of p47phox to activate the phagocyte NADPH oxidase. J Biol Chem. 2000;275:13793–801. 10788501 10.1074/jbc.275.18.13793

[83] Cocco T, Di Paola M, Papa S, Lorusso M. Arachidonic acid interaction with the mitochondrial electron transport chain promotes reactive oxygen species generation. Free Radic Biol Med. 1999;27:51–9. 10443919 10.1016/s0891-5849(99)00034-9

[84] Becknell B, Caligiuri MA. Interleukin-2, interleukin-15, and their roles in human natural killer cells. Adv Immunol. 2005;86:209–39. 15705423 10.1016/S0065-2776(04)86006-1

[85] Waldmann TA. The shared and contrasting roles of IL2 and IL15 in the life and death of normal and neoplastic lymphocytes: implications for cancer therapy. Cancer Immunol Res. 2015;3:219–27. 25736261 10.1158/2326-6066.CIR-15-0009 PMC4351780

[86] Hu X, Li J, Fu M, Zhao X, Wang W. The JAK/STAT signaling pathway: from bench to clinic. Signal Transduct Target Ther. 2021;6:402. 34824210 10.1038/s41392-021-00791-1 PMC8617206

[87] MacMullan MA, Wang P, Graham NA. Phospho-proteomics reveals that RSK signaling is required for proliferation of natural killer cells stimulated with IL-2 or IL-15. Cytokine. 2022;157:155958. 35841827 10.1016/j.cyto.2022.155958

[88] Stulnig TM, Huber J, Leitinger N, Imre EM, Angelisova P, Nowotny P, et al. Polyunsaturated eicosapentaenoic acid displaces proteins from membrane rafts by altering raft lipid composition. J Biol Chem. 2001;276:37335–40. 11489905 10.1074/jbc.M106193200

[89] Ma DW, Seo J, Davidson LA, Callaway ES, Fan YY, Lupton JR, et al. n-3 PUFA alter caveolae lipid composition and resident protein localization in mouse colon. FASEB J. 2004;18:1040–2. 15084525 10.1096/fj.03-1430fje

[90] Chen W, Jump DB, Esselman WJ, Busik JV. Inhibition of cytokine signaling in human retinal endothelial cells through modification of caveolae/lipid rafts by docosahexaenoic acid. Invest Ophthalmol Vis Sci. 2007;48:18–26. 17197511 10.1167/iovs.06-0619 PMC1975816

[91] Wong SW, Kwon MJ, Choi AM, Kim HP, Nakahira K, Hwang DH. Fatty acids modulate toll-like receptor 4 activation through regulation of receptor dimerization and recruitment into lipid rafts in a reactive oxygen species-dependent manner. J Biol Chem. 2009;284:27384–92. 19648648 10.1074/jbc.M109.044065 PMC2785667

[92] Varshney P, Yadav V, Saini N. Lipid rafts in immune signalling: current progress and future perspective. Immunology. 2016;149:13–24. 27153983 10.1111/imm.12617 PMC4981613

[93] Vamosi G, Bodnar A, Vereb G, Jenei A, Goldman CK, Langowski J, et al. IL-2 and IL-15 receptor alpha-subunits are coexpressed in a supramolecular receptor cluster in lipid rafts of T cells. Proc Natl Acad Sci U S A. 2004;101:11082–7. 15263076 10.1073/pnas.0403916101 PMC503744

[94] Pillet AH, Lavergne V, Pasquier V, Gesbert F, Theze J, Rose T. IL-2 induces conformational changes in its preassembled receptor core, which then migrates in lipid raft and binds to the cytoskeleton meshwork. J Mol Biol. 2010;403:671–92. 20816854 10.1016/j.jmb.2010.08.056

[95] Salavessa L, Lagache T, Malarde V, Grassart A, Olivo-Marin JC, Canette A et al. Cytokine receptor cluster size impacts its endocytosis and signaling. Proc Natl Acad Sci U S A. 2021;118. 10.1073/pnas.2024893118 PMC8449393 34504012

[96] Sehgal PB, Guo GG, Shah M, Kumar V, Patel K. Cytokine signaling: STATS in plasma membrane rafts. J Biol Chem. 2002;277:12067–74. 11815625 10.1074/jbc.M200018200

[97] Lanier LL. NKG2D receptor and its ligands in host defense. Cancer Immunol Res. 2015;3:575–82. 26041808 10.1158/2326-6066.CIR-15-0098 PMC4457299

[98] Serrano-Pertierra E, Cernuda-Morollon E, Brdicka T, Hooejsi V, Lopez-Larrea C. L-plastin is involved in NKG2D recruitment into lipid rafts and NKG2D-mediated NK cell migration. J Leukoc Biol. 2014;96:437–45. 24803550 10.1189/jlb.2A1013-564R PMC5395936

[99] Gutgemann SA, Sandusky MM, Wingert S, Claus M, Watzl C. Recruitment of activating NK-cell receptors 2B4 and NKG2D to membrane microdomains in mammalian cells is dependent on their transmembrane regions. Eur J Immunol. 2015;45:1258–69. 25545687 10.1002/eji.201444741

[100] Serrano-Pertierra E, Lopez-Larrea C, Using NK. Cell lipid raft fractionation to understand the role of lipid rafts in NK cell receptor signaling. Methods Mol Biol. 2016;1441:131–9. 27177662 10.1007/978-1-4939-3684-7_11

[101] Kandarian F, Sunga GM, Arango-Saenz D, Rossetti MA. Flow Cytometry-based cytotoxicity assay for the Assessment of Human NK Cell activity. J Vis Exp. 2017. 10.3791/56191 PMC5614136 28829424

[102] Zhou Y, Xu Y, Chen L, Xu B, Wu C, Jiang J. B7-H6 expression correlates with cancer progression and patient’s survival in human ovarian cancer. Int J Clin Exp Pathol. 2015;8:9428–33. 26464699 PMC4583931

