# Supplementary material for: Arachidonic acid impairs natural killer cell functions by disrupting signaling pathways driven by activating receptors and reactive oxygen species
Source: Cell Commun Signal. 2024 Nov 19;22:555. doi: 10.1186/s12964-024-01940-z (PMC11575453; doi:10.1186/s12964-024-01940-z)
Supplement: Supplementary file 2 — Supplementary Material 2 [file 12964_2024_1940_MOESM2_ESM.pdf]

Supplementary Figures

Arachidonic Acid Impairs Natural Killer Cell Functions by Disrupting Signaling Pathways driven by activating receptors and ROS

Mohamad K. Hammoud[1], Celina Meena[2], Raimund Dietze[1], Nathalie Hoffmann[2], Witold Szymanski[3,4], Florian Finkernagel[1,5], Andrea Nist[6], Thorsten Stiewe[6], Johannes Graumann[3,4], Elke Pogge von Strandmann[2] and Rolf Müller[1]

[1]Department of Translational Oncology, Center for Tumor Biology and Immunology, Philipps University, Marburg, Germany
[2]Institute of Tumor Immunology, Center for Tumor Biology and Immunology, Philipps University, Marburg, Germany
[3]Institute of Translational Proteomics, Biochemical Pharmacological Centre, Philipps University, Marburg, Germany
[4]Core Facility Translational Proteomics, Philipps University, Marburg, Germany
[5]Bioinformatics Core Facility, Philipps University, Marburg, Germany
[6]Genomics Core Facility, Philipps University, Marburg, Germany

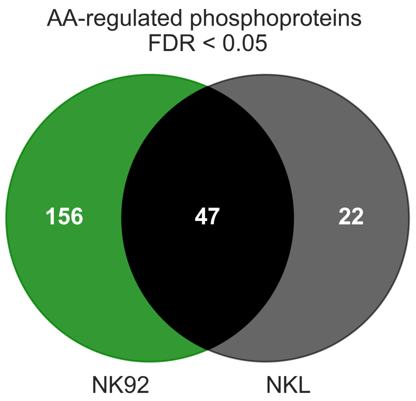

Figure 1. Venn diagram showing the overlap of proteins with regulated phosphosites in NK92 and NKL cells for significantly sites (FDR<0.05; see Fig. 1B for nominal p<0.05).

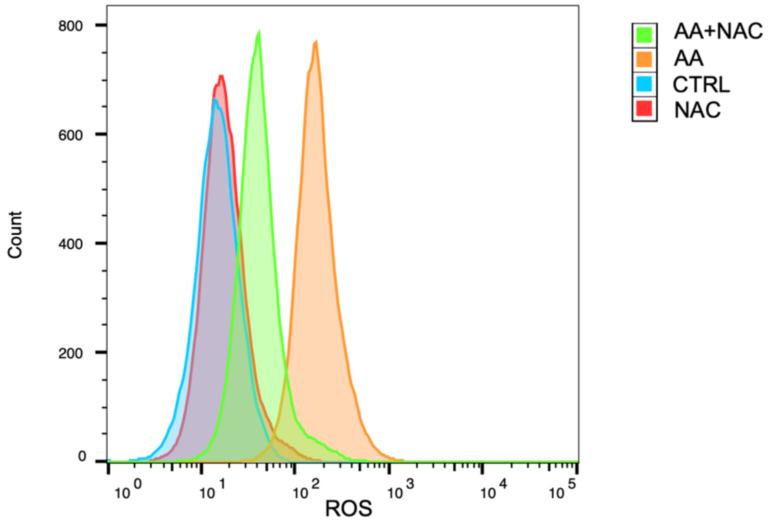

Figure S2. Representative histogram of flow-cytometry-based analysis of intracellular ROS levels in NK92 cells were treated with AA or solvent (CTRL) for 1 hr (quantification of n=4 replicates in Fig. 4D). The ROS scavenger NAC was included to verify the induction of ROS by AA.

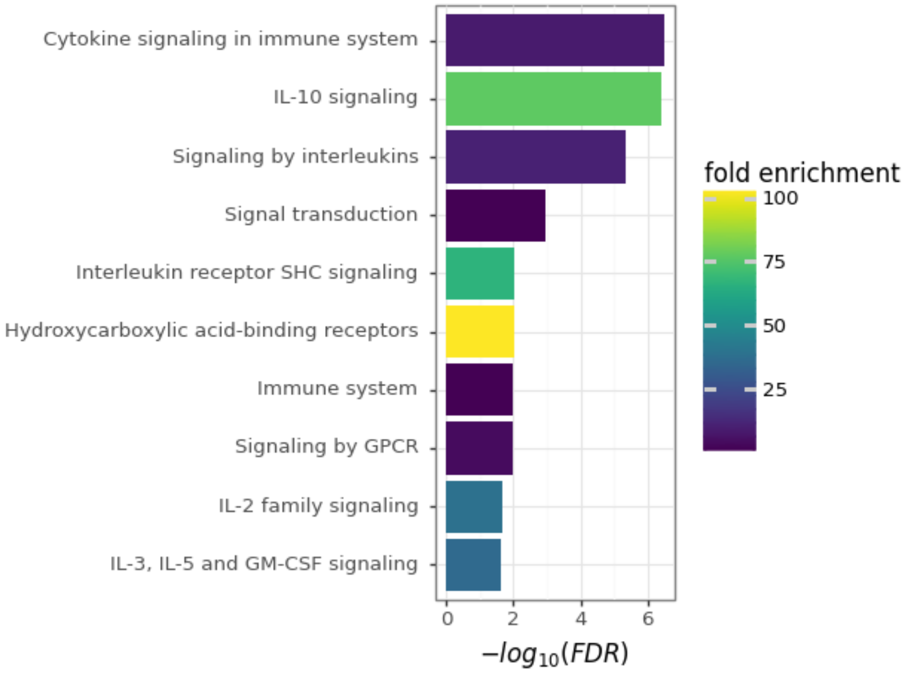

Figure S3. Functional annotation of the IL-2-induced genes repressed by AA using the online tool of the Database for Annotation, Visualization and Integrated Discovery (DAVID; https://david.ncifcrf.gov). The analysis included the n=69 genes (Fig. 4A).

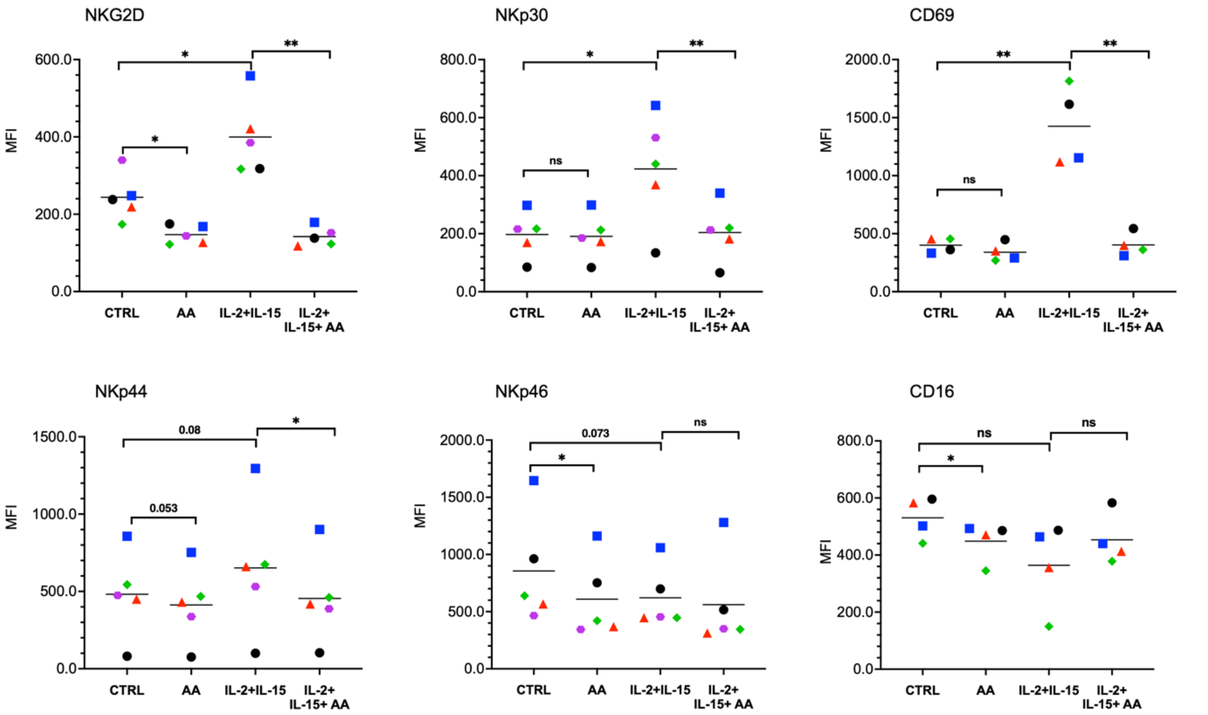

Figure S4. Flow-cytometric analysis of surface expression of the indicated NK cell receptors on primary NK cells treated with solvent (CTRL), AA, IL-2 plus IL-15 or AA plus IL-2 and IL-15 for 24 hrs. The plots show absolute MFI values (normalized data for n=4-5 replicates in Fig. 6A).

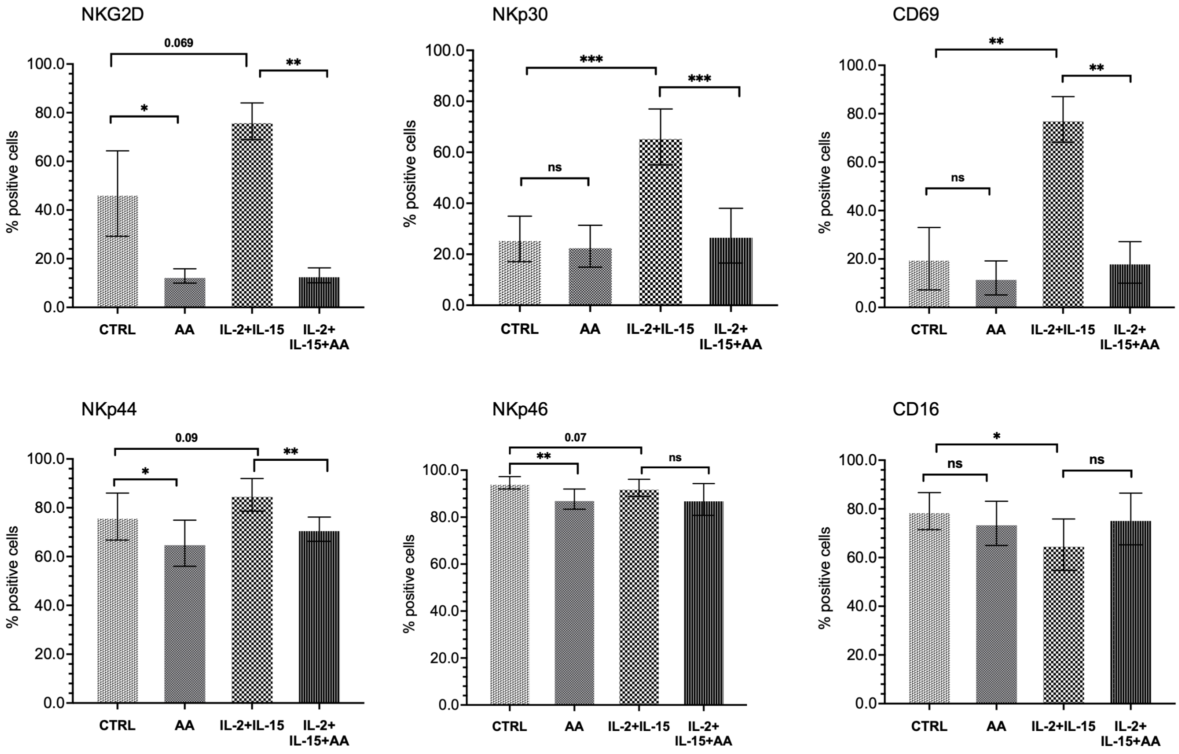

Figure S5. Flow-cytometric analysis of surface expression of the indicated NK cell receptors on primary NK cells treated with solvent (CTRL), AA, IL-2 plus IL-15 or AA plus IL-2 and IL-15 for 24 hrs. The plots show the percentage of positive cells in the analysis in Fig. 6A for n= 4-5 replicates. ***p < 0.001; **p< 0.01; *p< 0.05; ns: not significant by paired t test. P values close to the significance threshold are indicated as numbers.

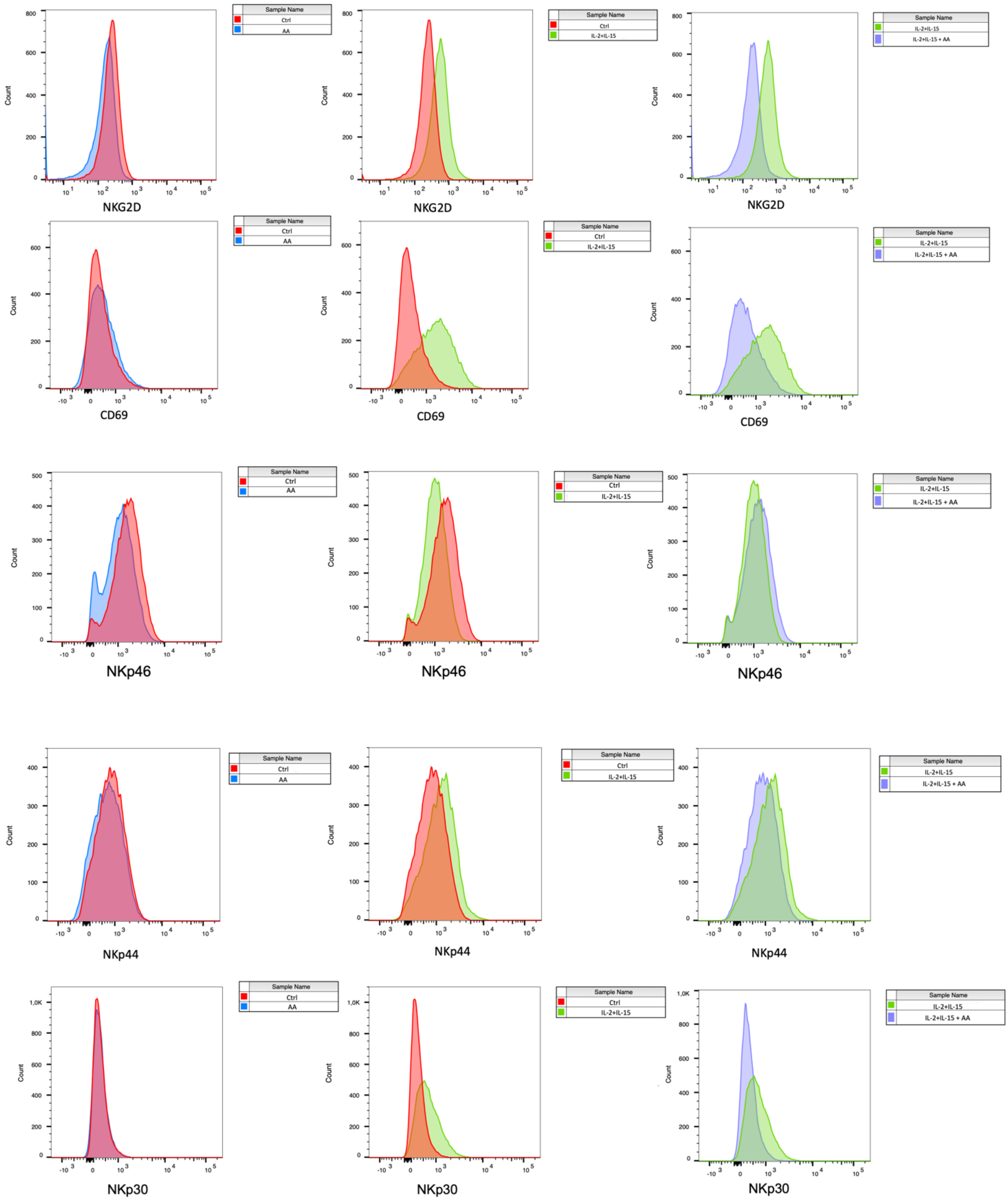

Figure S6. Histograms of the flow-cytometric analysis of the indicated NK cell receptors on primary NK cells treated with solvent (CTRL), AA, IL-2 plus IL-15 or AA plus IL-2 and IL-15 corresponding to the data in Fig. 6A.

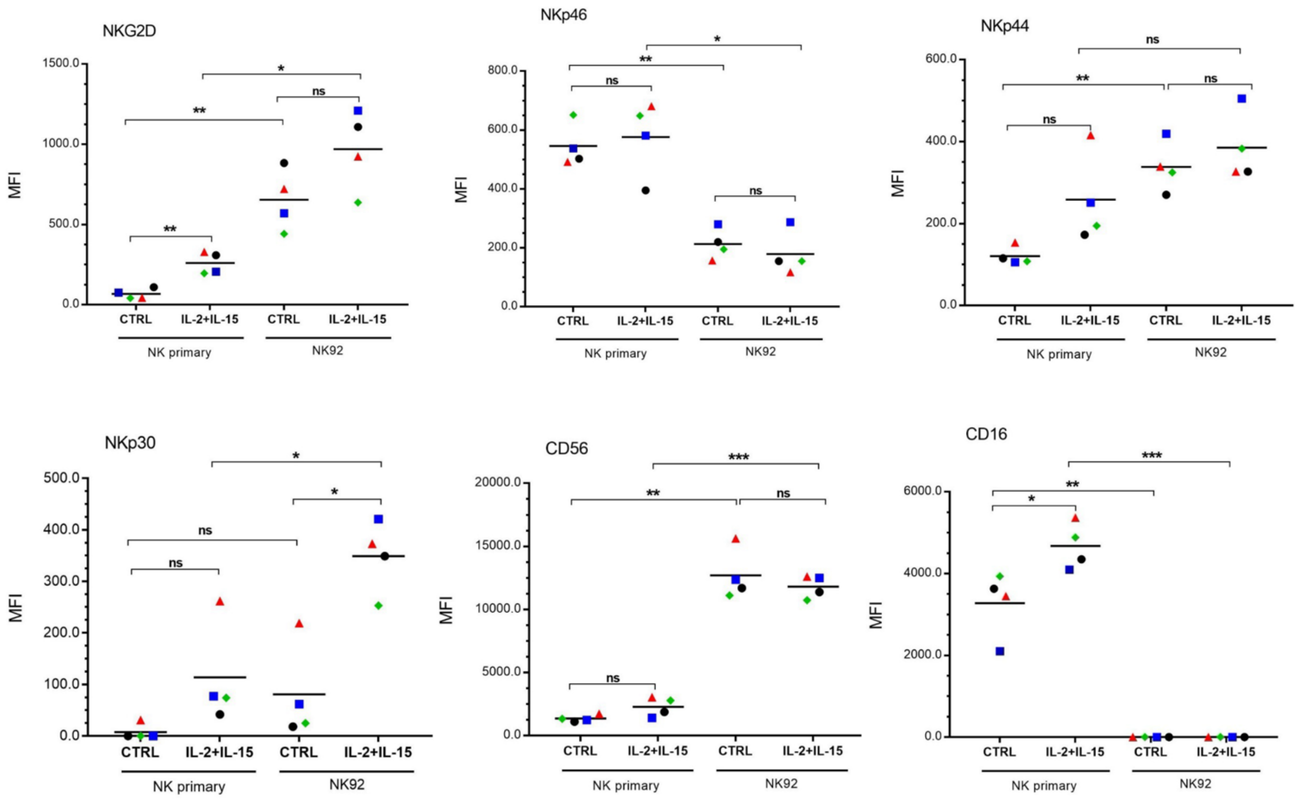

Figure S7. Flow-cytometric analysis showing the surface expression of NKG2D, NCRs, CD56 and CD16 on primary NK cells and NK92 cell line. Cells were incubated overnight with either IL-2 (10 U/ml; CTRL) or a combination of IL-2 (200 U/ml) and IL-15 (10 ng/ml) to induce the resting and activated states, respectively. The plots show the mean fluorescence intensity (MFI). Representative data of four independent replicates are displayed (n=4) and the mean values are indicated by horizontal bars. *p < 0.05; **p < 0.01; ***p < 0.001; ns: not significant by paired t-test.

The data show that receptors are NK cell receptors are expressed and NKG2D, NKp44 and NKp30 are induced upon stimulation in both models, indicating that NK92 are suitable as an experimental system in our study. As expected, the data also confirm that NK92 cells do not express CD16 and are positive for CD56.

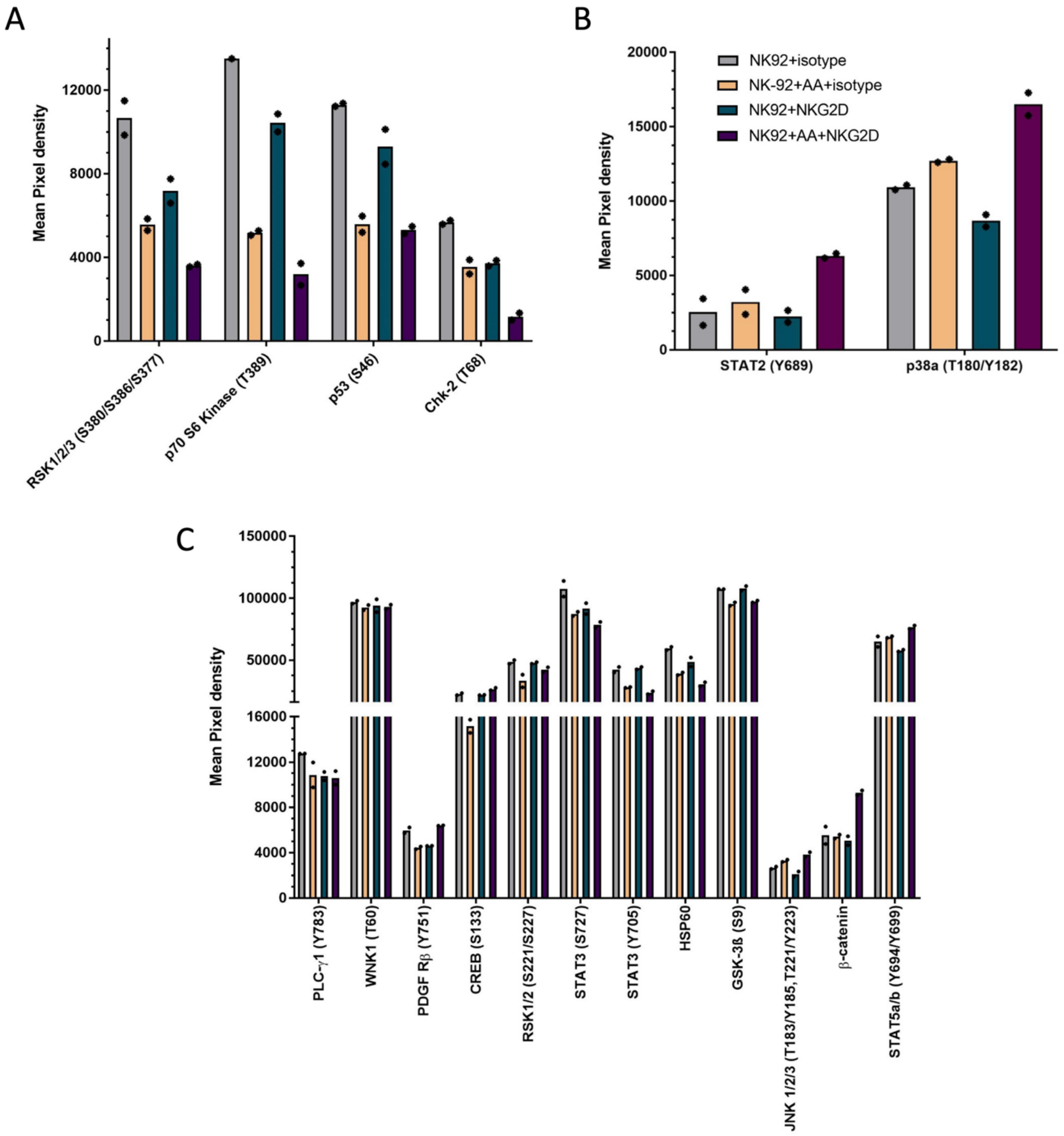

Figure S8. Effect of AA on the phosphorylation of signaling proteins upon antibody-mediated NKG2D engagement analyzed by the commercial Proteome Profiler Human Phospho-Kinase Array Kit. Experimental conditions were as described in the legend to Fig. 6D. Symbols show technical duplicates; bar represent the respective mean. **(A)** Sites repressed by NKG2D and AA; **(B)** sites unresponsive to NKG2D but upregulated by AA; **(C)** sites unresponsive to both NKG2D and AA.

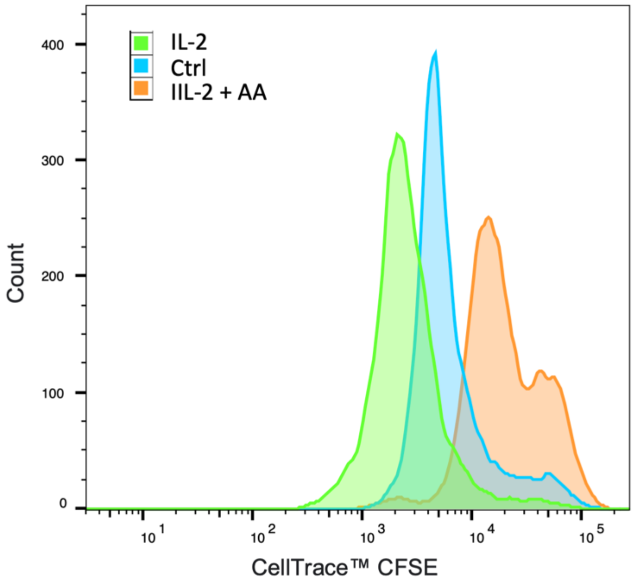

Figure S9. Representative histogram showing AA-mediated inhibition of IL-2-dependent NK92 cell proliferation. Cells were stained with CellTrace™ CFSE and analyzed by flow cytometry after 3 days. The plot shows the number of CFSE-positive cells for each condition. For details and a quantification of n=4 biological replicates see Fig. 8B.

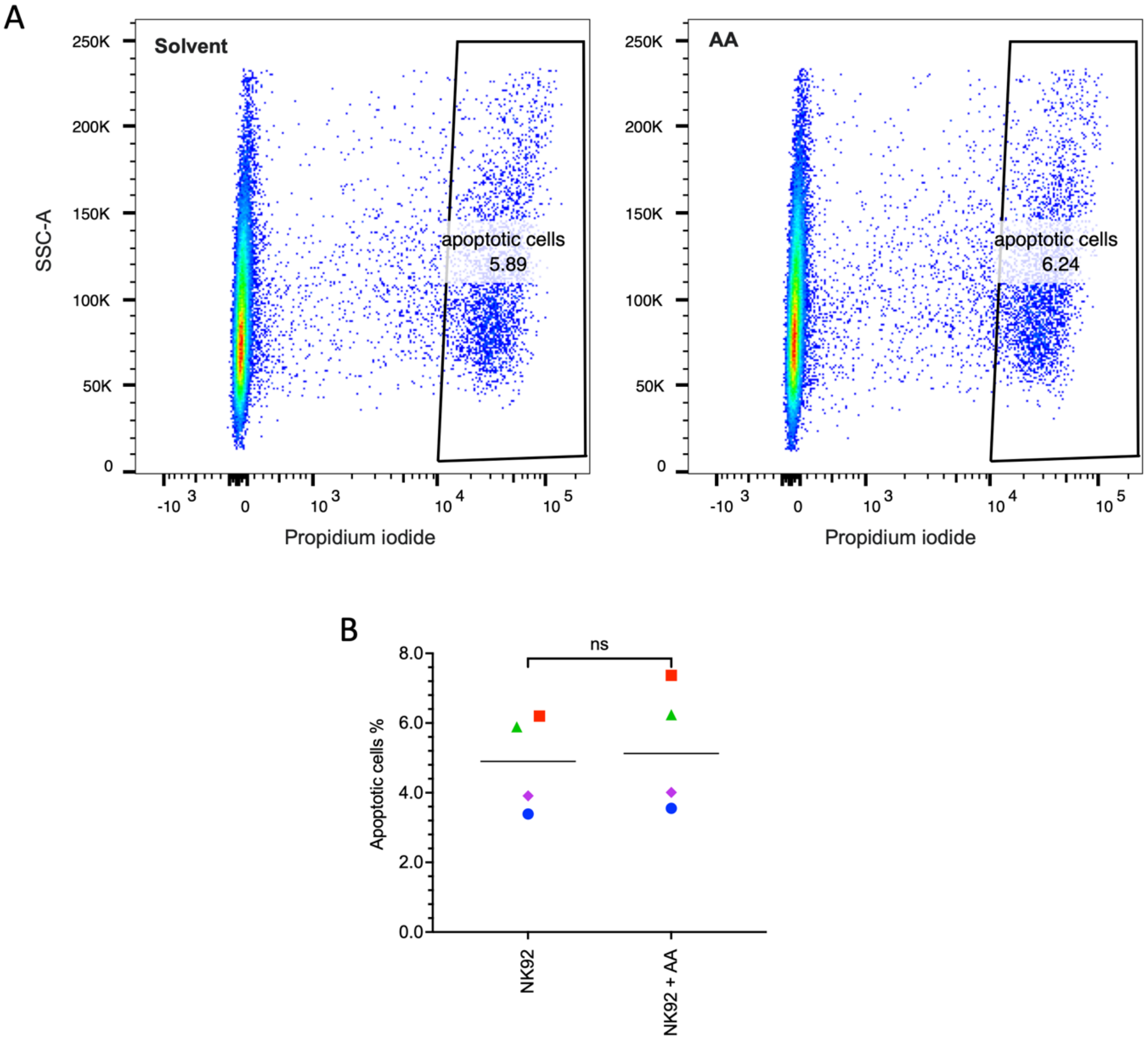

Figure S10. Propidium iodide staining of untreated and AA-treated NK92 cells. Cells were treated for 3 hrs and analyzed by flow cytometry. **(A)** Representative scatter plots. **(B)** Quantification of n=4 biological replicates. ns: not significant by paired t test. The data show that AA does not induce apoptosis of NK92 cells.

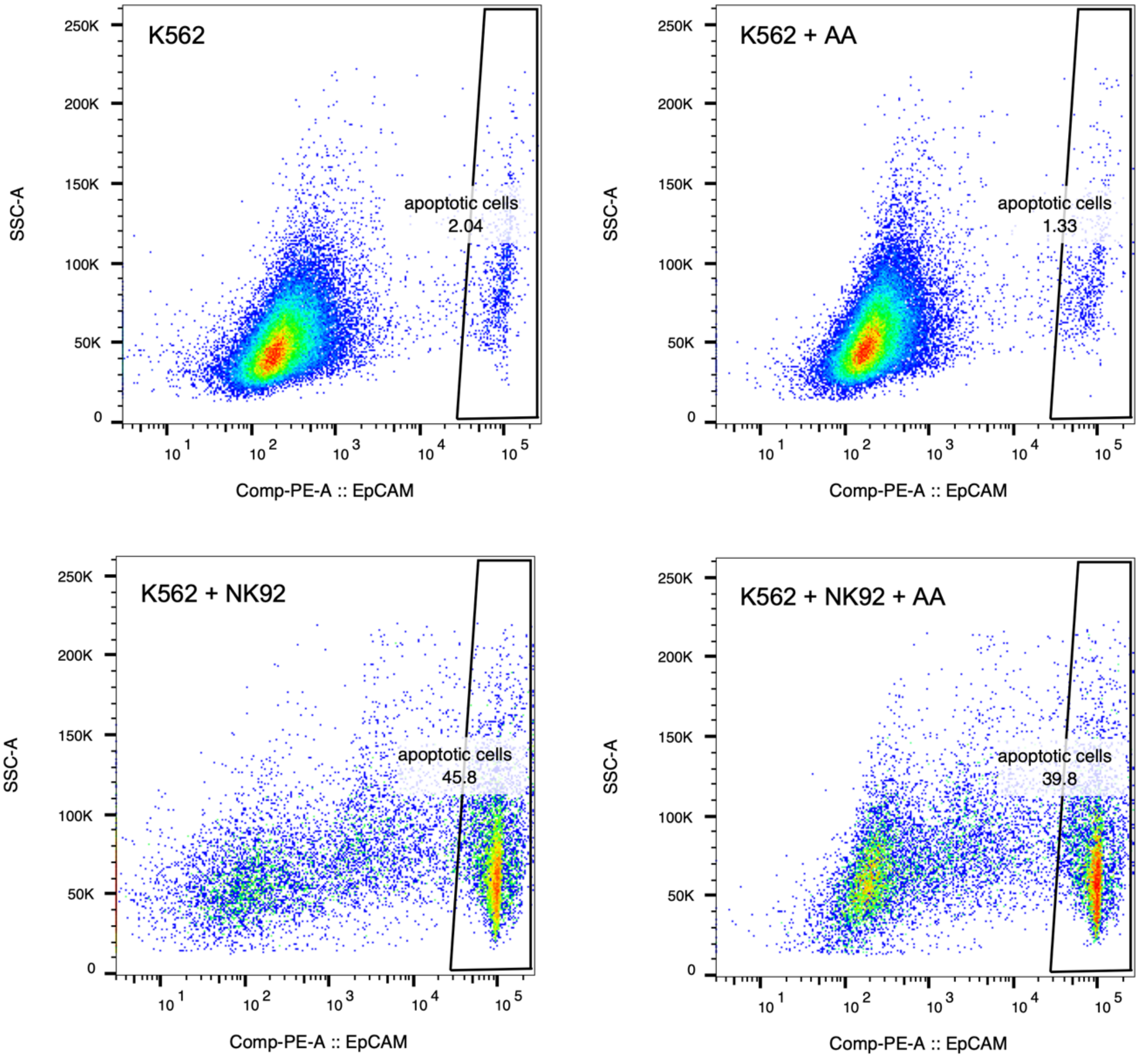

Figure S11. Representative scatter plots showing the percentage of apoptotic PI-positive K562 cells after incubation with NK92 (ratio 1:4) for 3 hrs. Cells treated for 3 hrs were analyzed by flow cytometry and gated for K562 cells. A quantification of n=4 biological replicates in shown in Fig. 8C.
